# Analysis of the co-operative interaction between the allosterically regulated proteins GK and GKRP using tryptophan fluorescence

**DOI:** 10.1042/BJ20131363

**Published:** 2014-04-11

**Authors:** Bogumil Zelent, Anne Raimondo, Amy Barrett, Carol W. Buettger, Pan Chen, Anna L. Gloyn, Franz M. Matschinsky

**Affiliations:** *Department of Biochemistry and Biophysics and Institute for Diabetes, Obesity and Metabolism, Perelman School of Medicine, University of Pennsylvania, Philadelphia, PA, U.S.A.; †Oxford Centre for Diabetes Endocrinology & Metabolism, University of Oxford, Oxford, U.K.

**Keywords:** allosteric regulation, co-operativity, diabetes, fructose phosphate ester, glucokinase, glucokinase regulatory protein, metabolic regulation, F1P, fructose 1-phosphate, F6P, fructose 6-phosphate, GCK/GK, glucokinase, GCKR/GKRP, GK regulatory protein, GKA, GK activator, GKRPI, GKRP inhibitor, hGK, human GK, hGKRP, human GKRP, MH, D-mannoheptulose, NATA, *N*-acetyl-L-tryptophanamide, NPC, nuclear pore complex, PPI, protein–protein interaction, rGKRP, rat GKRP, SIS, sugar isomerase, T2D, Type 2 diabetes, TF, tryptophan fluorescence, WT, wild-type, xGK, *Xenopus* GK, xGKRP, *Xenopus* GKRP

## Abstract

Hepatic glucose phosphorylation by GK (glucokinase) is regulated by GKRP (GK regulatory protein). GKRP forms a cytosolic complex with GK followed by nuclear import and storage, leading to inhibition of GK activity. This process is initiated by low glucose, but reversed nutritionally by high glucose and fructose or pharmacologically by GKAs (GK activators) and GKRPIs (GKRP inhibitors). To study the regulation of this process by glucose, fructose-phosphate esters and a GKA, we measured the TF (tryptophan fluorescence) of human WT (wild-type) and GKRP-P446L (a mutation associated with high serum triacylglycerol) in the presence of non-fluorescent GK with its tryptophan residues mutated. Titration of GKRP-WT by GK resulted in a sigmoidal increase in TF, suggesting co-operative PPIs (protein–protein interactions) perhaps due to the hysteretic nature of GK. The affinity of GK for GKRP was decreased and binding co-operativity increased by glucose, fructose 1-phosphate and GKA, reflecting disruption of the GK–GKRP complex. Similar studies with GKRP-P446L showed significantly different results compared with GKRP-WT, suggesting impairment of complex formation and nuclear storage. The results of the present TF-based biophysical analysis of PPIs between GK and GKRP suggest that hepatic glucose metabolism is regulated by a metabolite-sensitive drug-responsive co-operative molecular switch, involving complex formation between these two allosterically regulated proteins.

## Abstract 2

Using tryptophan fluorescence we explore in a unique way the co-operative interaction between two allosterically regulated proteins (GK and GKRP), which operate as a critical drug-sensitive molecular switch in hepatic carbohydrate metabolism.

## INTRODUCTION

The hexokinase GK (glucokinase) plays a critical role in the regulation of hepatic glucose metabolism [1–4]. It has a relatively low affinity for glucose (approximately 7.5 mM), allowing it to adjust its activity precisely in response to physiological changes in blood and intrahepatic glucose concentrations. This enables effective clearance of glucose from the blood after a meal. In contrast with other hexokinases, GK displays a sigmoidal activity curve with regard to glucose and is not inhibited by its product, glucose 6-phosphate, or other metabolites [4,5]. Approximately 99.9% of the body's entire supply of GK resides in the liver, with the remainder expressed in the endocrine cells of the pancreas, enteroendocrine cells, pituitary gonadotropes and selected nuclei of the central nervous system [3].

Gene expression and post-translational regulation of GK are profoundly influenced by its location in the body. In the liver its expression is effectively controlled by insulin such that absence of this hormone results in near total loss of GK expression within a few days [1,2,6–9]. Its enzymatic activity is also regulated within minutes by binding of the liver-specific regulatory protein GKRP (GK regulatory protein) [6–9]. GKRP is present in liver cells in a 2–3-fold molar excess compared with GK, and its expression is relatively independent of food intake and hormonal status. In complexing with GK, GKRP performs at least two functions: first, it serves as a cytosolic chaperone, allowing entry of GK into the nuclear space via the NPC (nuclear pore complex) [10,11]; and secondly, it creates an inactive nuclear pool of GK that can be readily released in response to changes in hepatic glucose or fructose levels. [1,2,6–11]. Cytosolic GK–GKRP protein complex assembly and nuclear trafficking are also modulated by phosphorylated hexose metabolites. Glucose and F1P (fructose 1-phosphate), a product of fructose and sorbitol metabolism, oppose GK–GKRP complex formation, nuclear sequestration and subsequent inhibition of GK activity, whereas F6P (fructose 6-phosphate), an intermediate of glycolysis, glycogenolysis and gluconeogenesis, counters these actions, at least in humans [12,13]. GKAs (GK activators) [3,14–21] and GKRPIs (GKRP inhibitors) [22], novel classes of drugs with potential as anti-diabetic agents, also disrupt the GK–GKRP complex, thus enhancing hepatic glucose uptake [3,19–22]. The exit of free GK from the nucleus is independent of GKRP and is mediated by the enzyme's nuclear export signal [10,11].

To understand these complex liver-specific regulatory mechanisms, it is necessary to account, at least semi-quantitatively, for the participating cellular compartments, i.e. the cytosolic and nuclear spaces. The relative nuclear volume of the hepatocyte is approximately 5% in the fed state, and may increase to 10–15% after extended starvation [1,23,24]. The distribution of GK between these two compartments is known to differ markedly in the fed compared with the fasting state; it is primarily cytosolic in the former and primarily nuclear in the latter [1,8–11]. In contrast, GKRP resides almost exclusively in the nuclear space. In fact, it is difficult to detect GKRP in the cytosol via routine histochemical methods, regardless of nutritional status. Model calculations based on an assumed increase in relative nuclear volume during fasting from 5% to 10% or even 15% [1,23,24], illustrate that the cytosolic and nuclear concentrations and ratios of these two interacting proteins undergo dramatic changes during regular or imposed feeding–fasting cycles. For example, assuming an equal concentration of GK in the two compartments in the fed state, the total cytosolic amount of free active enzyme would be approximately 20-fold higher that in the nucleus, and the GK/GKRP ratio would probably be high, approaching a factor of 5–10. Alternatively, assuming 66% nuclear sequestration of total GK during fasting would result in a 10–20-fold increase in the nuclear concentration of GK and a subsequent increase in the nuclear GK/GKRP ratio from approximately 1:50 to approximately 1:4, still guaranteeing near complete inhibition of nuclear GK activity by GKRP. These considerations have implications for our understanding of the GK–GKRP interaction, and are taken into account in the design of the present investigation (see below).

**Figure 1:**
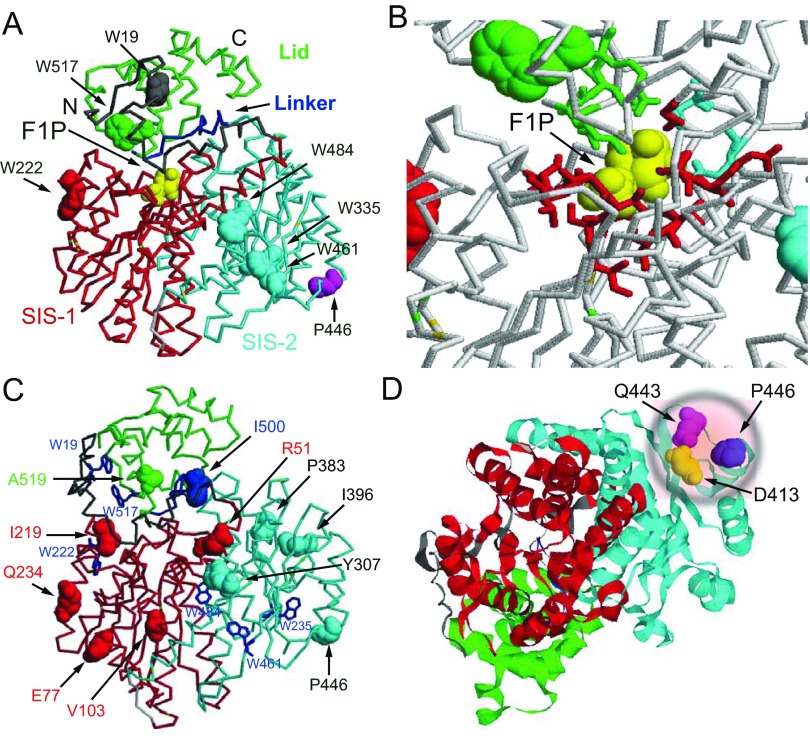
Structure of GKRP (**A**) Locations of tryptophan residues in GKRP domains: Trp^19^ at the N-terminus (6–44; grey), Trp^222^ in the SIS-1 domain (45–284; red), Trp^335^, Trp^461^ and Trp^483^ in the SIS-2 domain (289–499; cyan), and Trp^517^ in the Lid (513–606; green). The linker between SIS-1 and the Lid is shown in blue. The P446L point mutation is shown in magenta. F1P bound to one of the two known allosteric sites is shown in yellow. The second allosteric site for GKRPIs, situated between the Lid and the SIS-1–SIS-2 complex [22] is not shown. (**B**) Magnified view of the fructose phosphate-binding site to indicate the amino acids involved in F1P and F6P binding: Asn^512^, Lys^514^, Leu^515^ and Arg^518^ in the Lid (green), Glu^348^ and His^351^ in the SIS-2 domain (cyan), and Gly^107^, Thr^109^, Ser^110^, Glu^153^, Leu^178^, Ser^179^, Val^180^, Gly^181^, Leu^182^, Ser^183^, Gly^256^, Ser^258^ and Arg^259^ in the SIS-1 domain (red) (PDB code 4BB9). (**C**) The widely dispersed location of functional variants associated with triacylglycerol levels in GKRP. (**D**) The close proximity of the common mutant P446L to Asp^413^ [47] and Gln^443^ [49] forming a critical salt bridge to Arg^186^ of GK is illustrated.

The critical role of GK and its regulation by GKRP are illustrated by the impact that genetic variation in the genes that encode these proteins have on normal glucose and triacylglycerol metabolism in humans [25–32]. Over 600 naturally occurring mutations in the *GCK* (glucokinase) gene have been described, which have a wide range of functional consequences including inactivation or activation of catalytic capacity, structural and functional protein instability and decreased responsiveness to GKRP [29]. Heterozygous inactivating *GCK* mutations cause an autosomal dominantly inherited condition characterized by mild fasting hyperglycaemia, whereas inheritance of two defective *GCK* alleles results in the more severe phenotype of permanent neonatal diabetes [25,27]. In contrast, heterozygous activating mutations cause the opposite phenotype of hypoglycaemia, whereas homozygous cases have not been found because they are probably lethal [26]. A common non-synonymous variant (P466L) in *GCKR*, the gene that encodes GKRP, present in the healthy population has been reproducibly associated with fasting serum triacylglycerol and glucose levels [33]. Functional characterization of this variant protein has demonstrated that it is a less effective inhibitor of GK and results in reduced nuclear storage of GK [28,31]. Moreover, rare variants in *GCKR* have been shown to be associated with serum triacylglycerol and cholesterol levels in healthy adults and to be overrepresented in individuals with hypertriglyceridaemia (Figure 1C) [30,32].

In light of the central role of the GK–GKRP complex in glucose homoeostasis and its subsequent causal involvement in the pathogenesis of T2D (Type 2 diabetes), hypoglycaemia and hypertriglyceridaemia, it is not surprising that intensive efforts have been directed towards developing pharmacological agents that target both proteins [3,14–16,18–21,22,34]. These efforts have resulted in the discovery of GKAs a decade ago [15] and of GKRPIs in 2013 [22], which have an as yet unrealized potential as anti-diabetic agents. These pharmacological agents are of interest to the present investigation because of their capacity to disrupt the GK–GKRP complex, thereby indirectly enhancing glucose uptake and glycogen synthesis and also, potentially, lipid biosynthesis via GK activation.

Since the discovery of the GK and GKRP proteins, our understanding of the structural and functional biophysics of GK and GKRP and the manner in which they interact has steadily been advanced [1–7,9,35–46]. Kinetic, crystallographic and NMR studies have demonstrated that GK displays co-operative kinetics with respect to glucose (as indicated by a Hill coefficient of approximately 1.7), operates as a monomer, and exhibits a continual oscillation between low and high affinity conformations at a rate that is lower than that of the catalytic cycle, commonly referred to as hysteresis [36,44–46]. Biophysical characterization of GKRP and its interaction with GK has developed more slowly [9,19–21], but has recently benefited from the publication of several landmark crystallographic studies that provide detailed structural information about this protein and its PPIs (protein–protein interactions) with GK (Figures 1A and 1B) [22,47–49]. GKRP (of human, rat and frog origin alike) was shown to be a monomer, with a trilobate structure resembling the structure of SISs (sugar isomerases), featuring deeply buried allosteric sites: one for fructose-phosphate esters [47–49] and the other for GKRPIs [22]. There is clear evidence for conformational changes within GKRP (as shown for the rat protein) upon the allosteric binding of fructose-phosphate esters qualitatively distinguishing between the effects of F1P and F6P [49]. Choi et al. [47] succeeded in crystallizing and characterizing the *Xenopus laevis* GK–GKRP complex. One should remember here that xGKRP (*Xenopus* GKRP) is functionally unresponsive to fructose-phosphate esters, apparently the result of a critical H350P substitution. Beck and Miller [49] crystallized and characterized a GK–GKRP complex of rat GKRP and human pancreatic GK showing the same structural features as observed for the complex of frog proteins. The crystallographic observations of Choi et al. [47] and of Beck and Miller [49] thus demonstrate that the common GKRP-binding site on GK is located within the flexible hinge region which links the smaller and larger lobes of the enzyme. In combination with mutational analyses of hGKRP (human GKRP) and GKA binding to hGK (human GK) using TF (tryptophan fluorescence) or enzyme kinetics [21], these studies provide compelling evidence that GKRP and GKA bind to spatially distinct receptors in the allosteric modifier region of GK.

Notwithstanding this significant body of established knowledge, it remains to be clarified exactly how the complex system regulating hepatic glucose phosphorylation operates during feeding–fasting cycles, in pathological states, including T2D and hypoglycaemia, and in response to pharmacological intervention. As discussed above, highly effective allosteric and co-operative mechanisms coupled with intricate compartmental controls have evolved to regulate this process. In the present investigation, we used TF as a means to study hGK–hGKRP complex assembly and disassembly [37,39,41,43,50,51]. GKRP has six highly conserved tryptophan residues at positions Trp^19^, Trp^222^, Trp^335^, Trp^461^, Trp^484^ and Trp^517^ [48,49], randomly distributed throughout its trilobate structure (Figure 1). We used both GK-WT and a non-fluorescent form of GK in which its three native tryptophan residues were mutated (GK-W99R/W167F/W257F) to explore the interaction between GK and hGKRP-WT and GKRP-P446L, using the average TF of GKRP as an indicator of its conformation in its free and bound forms [51].

## MATERIALS AND METHODS

### Commercial materials and preparation of recombinant proteins

NATA (*N*-acetyl-L-tryptophanamide), D-glucose and D-fructose 6-phosphate disodium salt were supplied by Sigma Chemical. D-fructose 1-phosphate sodium salt was supplied by Santa Cruz Biotechnology. The non-fluorescent GKA used in the present study was RO0274375-000 [21,50,51]. Generation of GST-tagged islet GCK-WT, FLAG-tagged WT (wild-type) and GCKR-P446L bacterial expression vectors has been described previously [28,52]. Point mutations were introduced into the GST-GCK vector using the QuikChange II site-directed mutagenesis kit according to the manufacturer's instructions (Stratagene). The tryptophan residue-free GK made for the present study was GK-W99R/W167F/W257F. All mutant constructs were verified by DNA sequencing. Recombinant human β-cell GK was subsequently expressed in *Escherichia coli* cells and purified as described previously [52,53]. The GST-fusion proteins were then cleaved with factor Xa and submitted to a second round of purification using Benzamidine Sepharose 6B (GE Healthcare) according to the manufacturer's instructions. Recombinant GK-W99R/W167F/W257F protein was expressed in significant amounts and was found to be essentially pure as indicated by the presence of a single band at 50 kDa via gel chromatography (results not shown). Recombinant human GKRP-WT and GKRP-P446L were also expressed in *E. coli* cells and purified to homogeneity as described previously [28].

### Absorption and fluorescence measurements

#### UV–visible absorption

Spectra were measured using a Hitachi PerkinElmer U-3000 spectrophotometer.

#### Fluorescence

Spectra were measured with a Fluorolog-3-21 Jobin-Yvon Spex Instrument SA equipped with a 450 W Xenon lamp for excitation and a cooled R2658P Hamamatsu photomultiplier tube for detection. For all measurements, 90° geometry was employed and 295 nm excitation wavelengths were used to observe fluorescence emission in the 300–500 nm range. For TF, spectra slit width was set to provide a bandpath of 3 nm for excitation and 3 nm for emission. Spectra were corrected for instrumental response using Spex instrument software. A thermostatically controlled cell holder maintained sample temperature at 20°C. A thermocoupler was used to measure sample temperature. The buffer used contained 20 mM phosphate, 50 mM KCl, 1 mM EDTA and 1 mM DTT (pH 7.2).

#### Quantum yields

Fluorescence quantum yields of tryptophan (Φ) were determined for GKRP-WT and GKRP-P446L using eqn (1) [54]:

(1) $$\Phi_{S}=\Phi_{R}\;\left(A_{R}/A_{S}\right)\;\left(F_{S}/F_{R}\right)\;\left(n_{S}^{2}/n_{R}^{2}\right)$$

where S and R refer to sample and reference standard respectively, *A*_R_ and *A*_S_ denote absorbance at the excitation wavelength, *F*_S_ and *F*_R_ denote the integral intensities of the recorded fluorescence spectra measured under identical instrument settings, and *n*_S_ and *n*_R_ are the refractive indices. Fluorescence quantum yields were determined relative to NATA (Φ=0.14) [55].

#### Titration curves for binding of GK-W99R/W167F/W257F to GKRP-WT or GKRP-P446L

Fluorescence data were obtained via the stepwise addition of the appropriate amount of non-fluorescent GK-W99R/W167F/W257F to GKRP in a fluorescence cuvette. TF spectra were measured for GKRP in the presence of increasing amounts of GK-W99R/W167F/W257F, and spectra intensities corrected according to the background fluorescence of GK-W99R/W167F/W257F alone under the same experimental conditions. The change in GKRP protein fluorescence during titration was also measured using time-dependent scans. A manual procedure was used such that a typical titration with 10–12 additions required 20–25 min to perform. The same buffer as described above was routinely used unless stated otherwise. The bandpath was 0.5 nm at 295 nm for excitation and 12 nm at 340 nm for emission. Data were corrected for experimental dilution and then fit to the Langmuir saturation function *F*={*F*_0_+(*F*_sat_−*F*_0_)}{[*x*]^*H*^/([*x*]^*H*^+*K*_D_^*H*^)}, also known as the Hill equation, where *H* is the Hill number and *x* was F6P, F1P, GK-W99R/W167F/W257F, GKA or D-glucose. For some graphical presentations the linear form of the Hill equation was used: log[*F*/(*F*_sat_−*F*)]=*H* log *x*−*H* log *K*_D_ [46].

#### Protein concentration

The molar concentration of proteins was determined from UV absorption measurements in a quartz cuvette using a pathlength of 1 cm and a molar absorption coefficient at 280 nm of 47900 M^−1^·cm^−1^ for GKRP-WT [19] and 18510 M^−1^·cm^−1^ for GK-W99R/W167F/W257F, which was calculated based on previously established values for GK-WT (35130 M^−1^·cm^−1^) [35] and tryptophan (5540 M^−1^·cm^−1^) [56].

### The kinetics of ligand-induced slow conformational transitions of GK

Kinetic traces were monitored by TF in a quartz cuvette using 295±0.5 nm for excitation and 340±5 nm for emission at a temperature range of 5–25°C. These slow transitions were initiated by adding MH (D-mannoheptulose) to a final concentration of 5 or 10 mM to an assay mixture containing ~1 μM GK and 20 μM GKA. MH is a competitive inhibitor of GK and was used because the TF transitions in its presence are slower than those observed with glucose, thus facilitating kinetic analyses. For GK-W99R/W257F, the activation process was initiated by adding GKA in the absence of MH. The temperature dependence of the fluorescence tracings (see Supplementary Figure S1 at http://www.biochemj.org/bj/459/bj4590551add.htm) was fitted and analysed using the Risefall exponential model [57]. From the Arrhenius relationship lnk=ln*A*−*E*_a_/*RT*, and based on the precepts of transition state theory [58], we obtained the activation energy (*E*_a_) and other thermodynamic parameters of ligand-induced GK activation: enthalpy (Δ*H*°; kJ/mol), entropy (Δ*S*°; kJ/molK) and free energy of activation (Δ*G*°; kJ/mol), which were expressed as Δ*H*°=*E*_a_−*RT*, Δ*S*°=*R*[ln*A* −1−ln(*k*_B_*T*/*h*)] and Δ*G*°=Δ*H*°−*T*Δ*S*° respectively, where *k*_B_ is the Boltzman constant, *h* is the Planck constant, *R* the gas constant and *T* the absolute temperature. Kinetic data of the transition and thermodynamic parameters for WT and mutant GK are recorded in Supplementary Table S1 (http://www.biochemj.org/bj/459/bj4590551add.htm).

### Precision estimates

The numerical values of all Tables are the results of individual experiments. In many, but not all, instances results were verified by additional measurements. This approach is state of the art for studies of the present type, but was also dictated by the scarcity of the recombinant proteins combined with the strategy of using a large variety of experimental conditions to explore GK–GKRP interaction in depth.

### Graphics

Structural considerations for GKRP are based on PDB code 4BB9 [48] and 4LC9 [49] and for GK on PDB codes 1V4T (open form) and 1V4S (closed form) [38] using the RasMol program (version 2.7.1).

## RESULTS AND DISCUSSION

### Conceptual and methodological considerations

This investigation is based on a conceptual model of the GK–GKRP interaction, which was first suggested by Shiota et al. [11] and Bosco et al. [10] and subsequently reviewed by Agius [1] and Iynedjian [2]. This model proposes that GKRP plays a dual role in the regulation of GK. First, it behaves as a chaperone for GK transfer into the nucleus, as GK itself appears to lack a functional NLS (nuclear localization signal). Secondly, GKRP retains GK in the nucleus in an inactive, but readily accessible, inhibitory complex. The critical step in GK–GKRP complex formation is investigated in the present paper biophysically by TF under conditions mimicking the cytosol, at GK/GKRP ratios ranging from 1:1 to 10:1. These TF studies were performed at micromolar protein concentrations similar to those that occur in the cytosol. Complex formation was monitored via detection of changes in GKRP TF in the presence of increasing amounts of non-fluorescent GK-W99R/W167F/W257F. The common disease-associated variant protein GKRP-P446L, which has been well characterized in biochemical, genetic and cell biological studies [28,30,31], was also used to explore the potential of these TF-based methods of analysis and provided the means to assess the contribution of structural instability and/or impaired protein folding to the effectiveness of GKRP as a GK regulator [50]. Further critical experimental material and conceptual commentary on the biochemical characteristics of GK-W99R/W167F/W257F are shown in the Supplementary Online Data to facilitate the interpretation of the present model studies on GK–GKRP interactions employing this non-fluorescent reagent (Supplementary Figures S1–S4 and Tables S1–S3 with associated text and discussion at http://www.biochemj.org/bj/459/bj4590551add.htm). This Supplementary Online Data shows that the modified GK molecule retains its unique biochemical features with one exception: the activation of GK-W99R/W167F/W257F by GKAs is rendered glucose-independent as a result of the Trp^99^ substitution by arginine which makes the allosteric drug receptor site of the enzyme freely accessible to GKAs.

### Assessment of the basic TF characteristics of recombinant hGKRP

**Figure 2:**
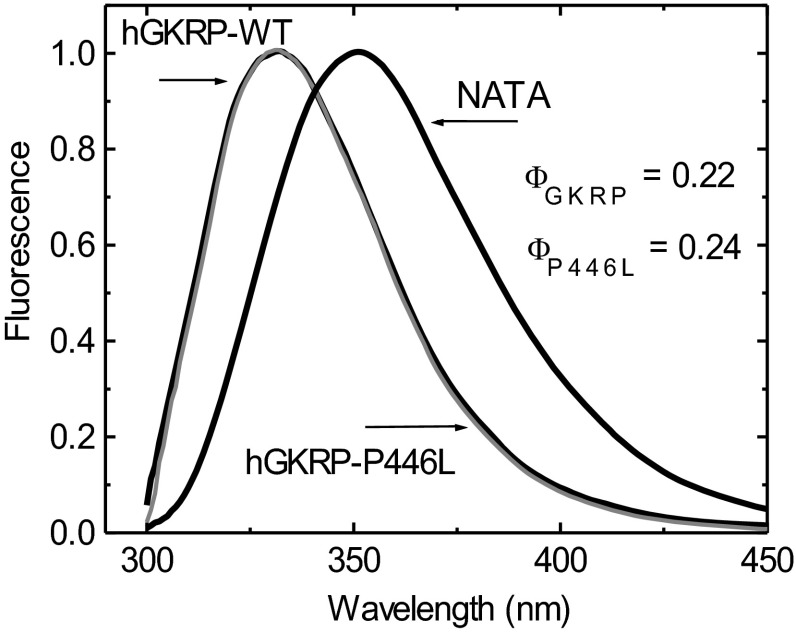
Normalized TF spectra for 0.3 μM GKRP-WT and GKRP-P446L compared with tryptophan–NATA (*λ*_exc_=295 nm)

**Figure 3:**
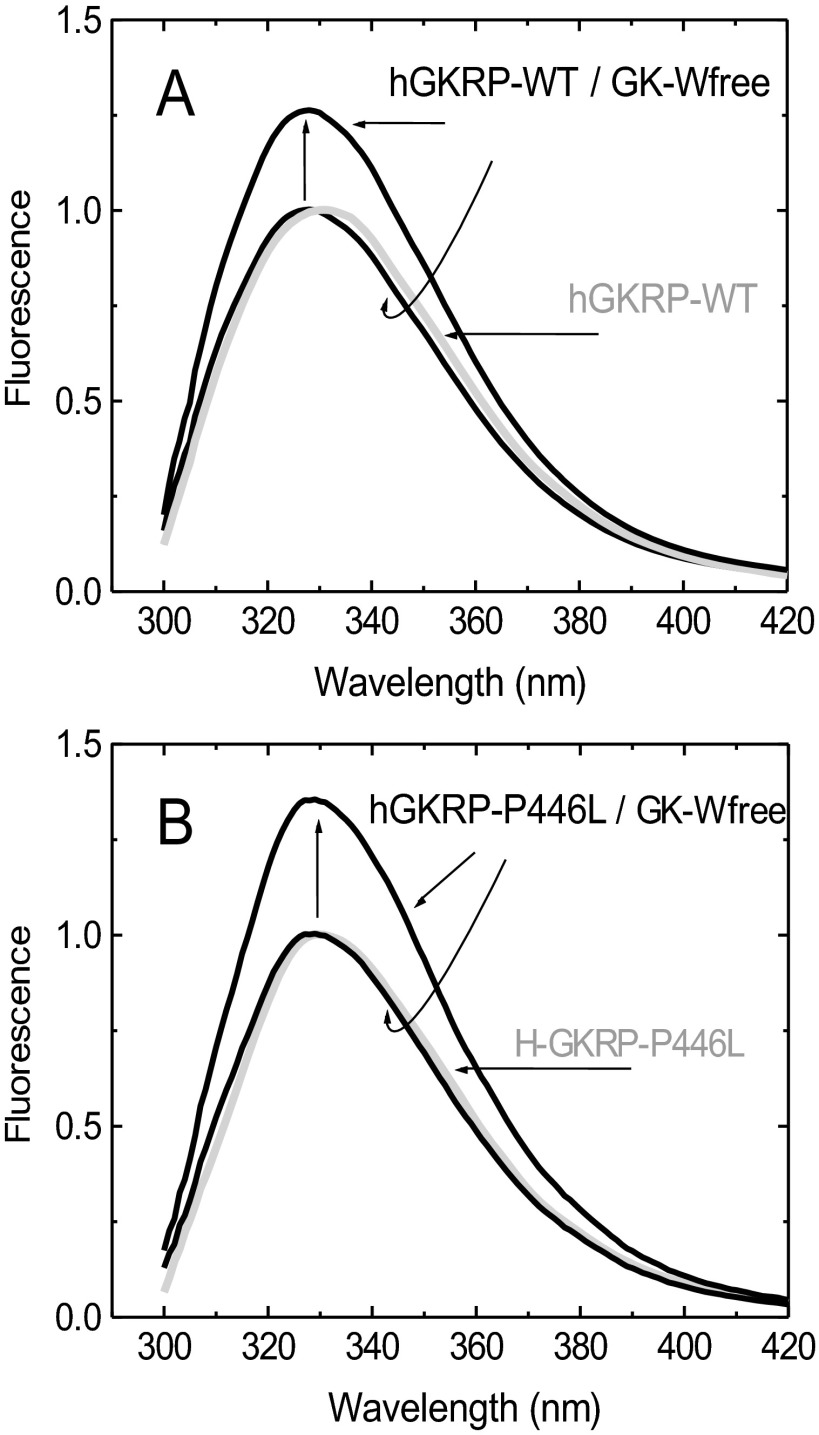
TF spectra for 0.3 μM GKRP in the presence or absence of 5 μM GK at 20°C showing the fluorescence increase (vertical arrows) and blue shift of the spectra upon GK binding for GKRP-WT (A) and GKRP-P446L (B) (*λ*_exc_=295 nm) GK-Wfree, GK-W99R/W167F/W257F.

The high average quantum yield of GKRP TF allowed for accurate measurements at 0.3 μM GKRP-WT and GKRP-P446L. The two proteins showed comparable spectra and quantum yields with the same TF maxima at 332 nm (Figures 2 and 3, and Supplementary Table S4 at http://www.biochemj.org/bj/459/bj4590551add.htm). Saturating amounts of GK (5 μM) increased the TF of GKRP-WT by 29% and GKRP-P446L by 44% (Figure 3 and Table 1). GK addition also caused a comparable blue shift in the TF maxima by 3–5 nm (Figure 3, and Supplementary Figures S5 and S6 at http://www.biochemj.org/bj/459/bj4590551add.htm). These results imply that GKRP undergoes a conformational change during complex assembly, which has been previously observed for the hGK–rGKRP (rat GKRP) complex crystallographically [49] and for the hGK–hGKRP complex by MD [47], but not with xGK (*Xenopus* GK) and xGKRP by crystallography [47]. Glucose, F6P, F1P and GKA had differential effects on the TF of GKRP-WT and GKRP-P446L. Glucose had no effect on the fluorescence of GKRP-WT, but lowered the TF signal observed for GKRP-P446L, suggesting that this amino acid substitution modifies the sugar phosphate-binding site of GKRP in a manner that specifically affects binding of unphosphorylated hexoses (Supplementary Figures S7 and S8 at http://www.biochemj.org/bj/459/bj4590551add.htm). An increase and decrease in the TF signal for both GKRP-WT and GKRP-P446L was observed in the presence of F6P and F1P respectively. Remarkably, positive as well as negative ΔTF values for GKRP-P446L were twice that of WT-GKRP for both phosphate esters (Table 1). Also noteworthy is the sigmoidal shape of the TF concentration-dependency curve for F6P contrasting with the hyperbolic shape of that for F1P as indicated by the different Hill coefficients. A corollary for these opposite effects of the two fructose-phosphate esters on GKRP TF are crystallographic observations showing that the Lid domain/SIS-2 interface of GKRP is differentially affected by the two ligands [49]. The TF changes encountered in the present study ranged in magnitude from 4 to 44% and therefore allowed for accurate ligand concentration-dependency studies, as well as assessment of the structural stability and refolding capacity of GKRP-WT and GKRP-P446L following denaturation by urea.

**Table 1:** GKRP-WT or GKRP-P446L interaction in the absence and presence of GK-W99R/W167F/W257F, F6P, F1P, D-glucose and GKA at 20°C measured by the TF of GKRP Δ*F* is the relative fluorescence change at 340 nm.

GKRP	Additional agents	Δ*F*	*S*_0.5_ (μM)	*H*
GKRP-WT	F6P	0.04	278	3.6
GKRP-P446L	F6P	0.07	242	2.94
GKRP-WT	F1P	−0.04	65	1
GKRP-P446L	F1P	−0.08	139	1
GKRP-WT	D-Glucose	0	–	–
GKRP-P446L	D-Glucose	−0.06	27 mM	1.49
GKRP-WT	GK-W99R/W167F/W257F	0.29	1.22	1.74
GKRP-P446L	GK-W99R/W167F/W257F	0.44	2.05	1.86
GKRP-WT	F6P and GK-W99R/W167F/W257F	0.31	1.20	1.71
GKRP-P446L	F6P and GK-W99R/W167F/W257F	0.44	1.66	1.31
GKRP-WT	F1P and GK-W99R/W167F/W257F	0.32	2.68	2.8
GKRP-P446L	F1P and GK-W99R/W167F/W257F	0.41	2.95	2.84
GKRP-WT	D-Glucose and GK-W99R/W167F/W257F	0.42	2.56	2.78
GKRP-P446L	D-Glucose and GK-W99R/W167F/W257F	0.43	3.38	2.94
GKRP-WT	GKA and GK-W99R/W167F/W257F	0.42	2.77	3.4
GKRP-P446L	GKA and GK-W99R/W167F/W257F	0.43	2.59	2.17
GKRP-WT	D-Glucose, F6P and GK-W99R/W167F/W257F	0.35	2.43	2.83
GKRP-P446L	D-Glucose, F6P and GK-W99R/W167F/W257F	0.42	2.84	2.45
GKRP-WT	D-Glucose, F1P and GK-W99R/W167F/W257F	0.33	3.3	2.25
GKRP-P446L	D-Glucose, F1P and GK-W99R/W167F/W257F	0.44	2.97	2.77
GKRP-WT	D-Glucose, GKA and GK-W99R/W167F/W257F	0.42	4.75	3.2
GKRP-P446L	D-Glucose, GKA and GK-W99R/W167F/W257F	0.42	3.55	2.68

### Assessment of structural stability and studies of unfolding/refolding cycles of GKRP-WT and GKRP-P446L

**Figure 4:**
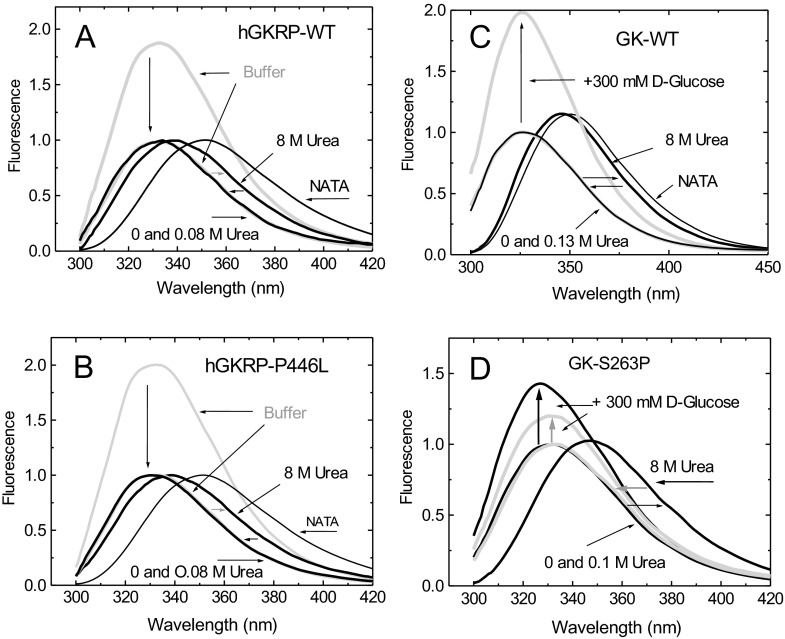
Structural stability and unfolding/refolding cycles of GKRP-WT and GKRP-P446L TF spectra for 0.3 μM GKRP in the presence or absence of 8 M urea at 20°C for GKRP-WT (**A**) and for GKRP-P446L (**B**). The horizontal arrows show the red and blue shifts of the spectra during denaturation and refolding at 4°C respectively. Vertical arrows show the TF spectra of the proteins in buffer normalized to 1. (**C**) TF spectra of WT-GK and (**D**) established instability mutant GK-S263P in the presence or absence of 8 M urea (*λ_e_*_xc_=295 nm).

A measure of the structural stability of GKRP-WT and GKRP-P446L, as well as the ability of these two proteins to refold spontaneously upon denaturation, were critical for the biophysical studies described in the present paper [50,59]. We found that GKRP-WT is a relatively labile protein, as indicated by a Δ*G*(H_2_O) of 1.56 kcal/mol, and is comparable with GK-WT (1.63 kcal/mol). Both GKRP-WT and GK-WT refold readily upon denaturation with 8 M urea (Figure 4 and Supplementary Table S5 at http://www.biochemj.org/bj/459/bj4590551add.htm). Furthermore, GKRP-WT and GKRP-P446L displayed comparable Δ*G*(H_2_O) of denaturation (1.56 and 1.55 kcal/mol respectively) and unfolding/refolding patterns, suggesting that structural instability is unlikely to explain the metabolic effects of this variant protein. This conclusion is strengthened by results obtained by comparison with the proven instability mutants GK-M298K and GK-S263P [59] which clearly showed a lowering of their Δ*G* of denaturation and impaired refolding after urea denaturation (Figure 4 and Supplementary Table S5).

### Demonstrating co-operativity in the assembly of the GK–GKRP complex

**Figure 5:**
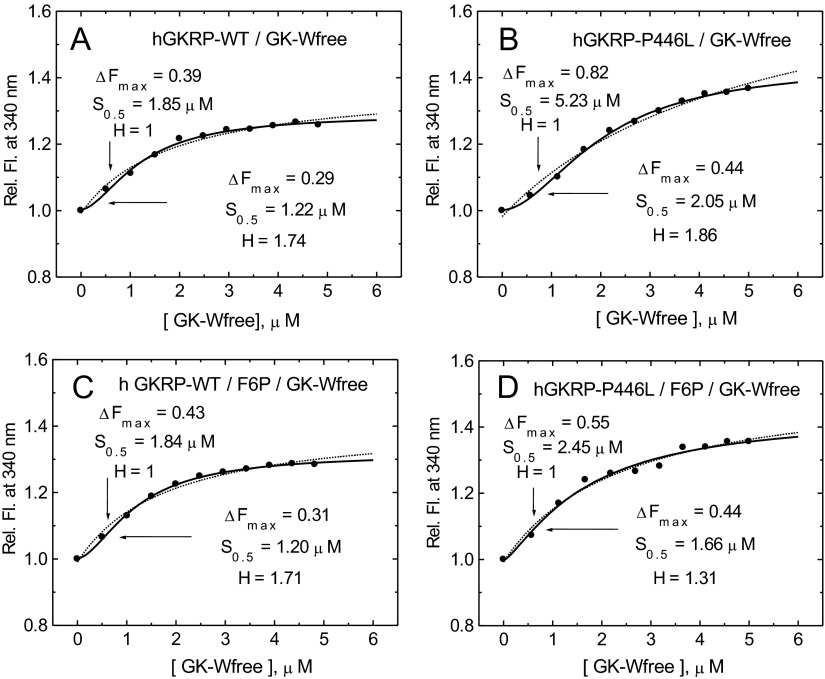
Co-operativity of GK–GKRP complex assembly (**A** and **B**) Relative TF increase for 0.3 μM GKRP in the presence of increasing amounts of GK-W99R/W167F/W257F (GK-Wfree) for GKRP-WT (**A**) and for GKRP-P446L (**B**). (**C** and **D**) The effect of increasing amounts of GK-W99R/W167F/W257F on the TF of 0.3 μM GKRP in the presence of 500 μM F6P are shown for GKRP-WT (**C**) and GKRP-P446L (**D**) (*λ*_exc_=295 nm; λ_em_=340 nm). Comparison of the curve fitting by simple saturation or sigmoidal functions is shown in Supplementary Table S6 (at http://www.biochemj.org/bj/459/bj4590551add.htm).

**Figure 6:**
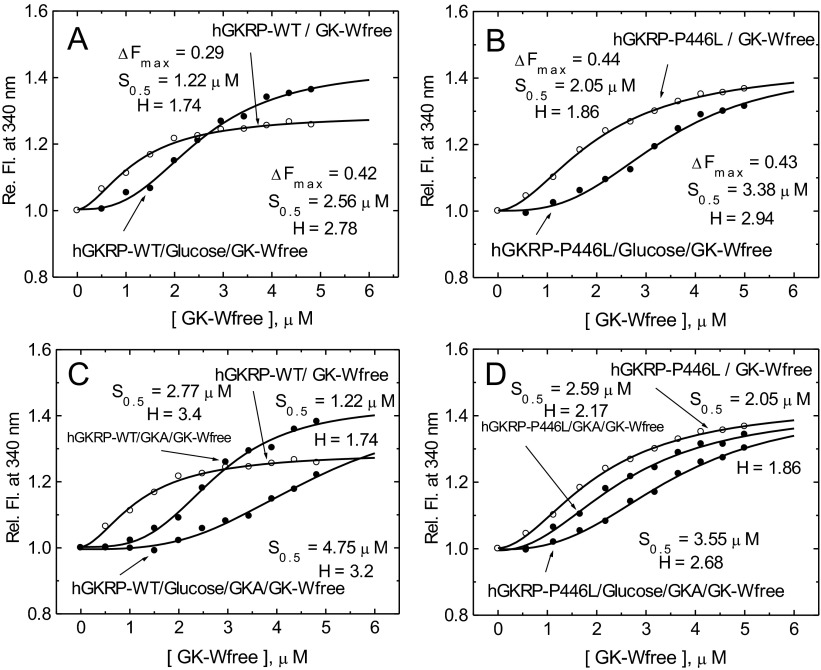
Co-operativity of GK–GKRP complex assembly (**A** and **B**) Relative TF change for 0.3 μM GKRP in the presence of increasing amounts of GK-W99R/W167F/W257F (GK-Wfree), and in the absence (○) or presence (●) of 100 mM D-glucose for GKRP-WT (**A**) and for GKRP-P446L (**B**). (**C** and **D**) The effect of increasing amounts of GK-W99R/W167F/W257F on the change in TF for 0.5 μM GKRP can be seen in the absence (○) or presence (●) of 20 μM GKA or 20 μM GKA and 100 mM D-glucose for GKRP-WT (**C**) and GKRP-P446L (**D**) (*λ*_exc_=295 nm; *λ*_em_=340 nm).

**Figure 7:**
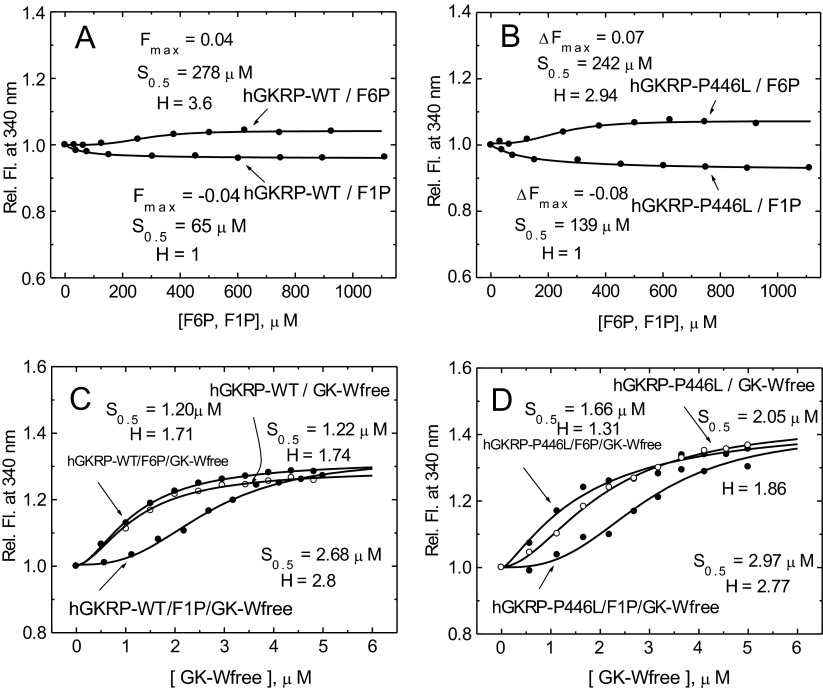
Fructose phosphate ester binding to GKRP and co-operativity of GK–GKRP complex assembly (**A** and **B**) Relative change in TF for 0.3 μM GKRP in the presence of increasing amounts of F6P and F1P for GKRP-WT (**A**) and GKRP-P446L (**B**). (**C** and **D**) The effect of increasing amounts of GK-W99R/W167F/W257F (GK-Wfree) on the change in TF for 0.5 μM GKRP-WT in the absence (○) or presence (●) of 500 μM F6P or 500 μM F1P for GKRP-WT (**C**) and for GKRP-P446L (**D**) (*λ*_exc_=295 nm; *λ*_em_=340 nm).

**Figure 8:**
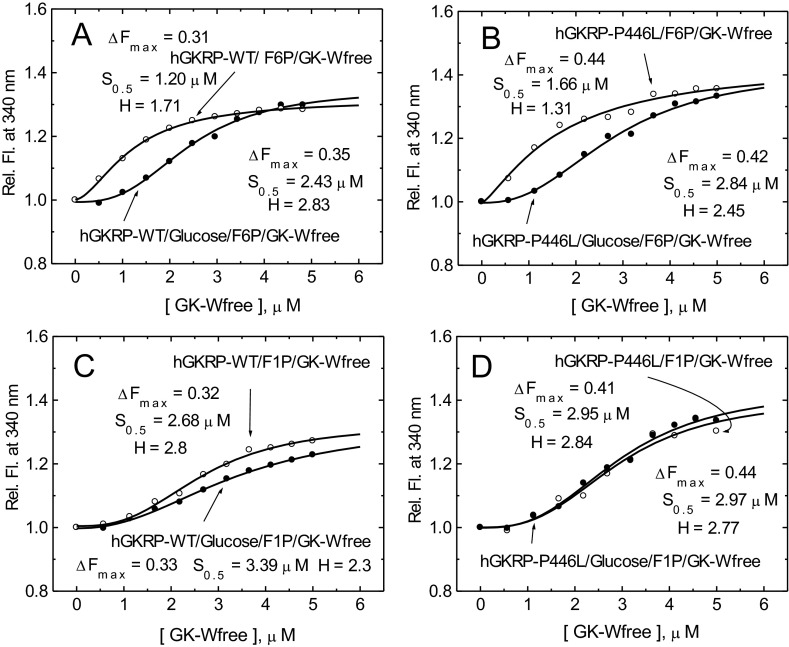
Co-operativity of GK–GKRP complex assembly (**A** and **B**) Relative change in TF for 0.3 μM GKRP in the presence of increasing amounts of GK-W99R/W167F/W257F (GK-Wfree) on the addition of 500 μM F6P (○) or 500 μM F6P and 100 mM D-glucose (●) for GKRP-WT (**A**) and GKRP-P446L (**B**). (**C** and **D**) The effect of increasing amounts of GK-W99R/W167F/W257F on the change in TF for 0.3 μM GKRP in the presence of 500 μM F1P (○) and 500 μM F1P and 100 mM D-glucose (●) can be seen for GKRP-WT (**C**) and GKRP-P446L (**D**) (*λ*_exc_=295 nm; *λ*_em_=340 nm).

In order to mimic the concentrations of GK and GKRP likely to occur in the cytosol, assembly of these two proteins was studied at a constant GKRP concentration of 0.3 μM with increasing amounts of GK ranging from 0.5 to 5 μM. Sigmoidal TF dose–response curves were observed with increasing amounts of GK-W99R/W167F/W257F, as indicated by indistinguishable Hill coefficients of 1.74 and 1.86 in the presence of GKRP-WT and GKRP-P446L respectively (Figures 5A and 5B, and Table 1). Saturating levels of F6P had no significant effect on the binding of GKRP-WT, but increased the affinity of GKRP-P446L for GK and lowered the degree of co-operativity of the PPI (*H*=1.31 compared with 1.86). This conclusion was supported by curve fits using simple saturation or sigmoidal functions (Figure 5 and Supplementary Table S6 at http://www.biochemj.org/bj/459/bj4590551add.htm). However, on average, the *S*_0.5_ value of GK for complex formation of approximately 1.2 μM in the presence of GKRP-WT was 35% lower than that obtained with GKRP-P446L (Figure 5). Saturating concentrations of glucose, F1P or GKA, either alone or in combination, markedly increased the sigmoidicity of these dose–response curves (Figures 6–8 and Table 1), indicating enhanced co-operativity. In the case of GKRP-WT the effect was most pronounced in the presence of high glucose and GKA, resulting in a 4-fold increase in the *S*_0.5_ value for GK and a 1.84-fold increase in the Hill coefficient (Figure 6C and Table 1). GKRP-P446L was less responsive to the combined action of saturating glucose and GKA, since the GK *S*_0.5_ value increased by only 1.73-fold and the Hill coefficient by 1.44-fold (Figure 6D and Table 1). Furthermore, the maximal ΔTF value of GKRP-P446L was generally constant at approximately 0.41–0.44 under all conditions tested, compared with a wider range of 0.29–0.42 for GKRP-WT, depending on the assay conditions. When titration of GKRP-WT with GK was performed in the presence of glucose, F1P or GKA, the TF dose–response curves were shifted to the right and corresponded to changes in the biophysical constants of these ligands largely consistent with known effects (Figures 6–8 and Table 1). The strikingly high efficacy of GKA alone (known not to activate unmodified GK in the absence of glucose [3]) is readily explained by the W99R substitution in GK, the effect of which would be to facilitate access of the GKA to its binding site ([50,51] and Supplementary Online Data). These qualitative and quantitative differences between GKRP-WT and GKRP-P446L are strikingly displayed in Figure 6. The co-operative nature of the GK–GKRP interaction and the extent to which this interaction is modified by physiological ligands and GKAs is further illustrated in Hill plots (Supplementary Figure S9 at http://www.biochemj.org/bj/459/bj4590551add.htm). Since the TF spectra of proteins are influenced by their basic structure and conformational rearrangements upon ligand binding, these results strongly suggest that the P446L substitution on GKRP results in an overall change in structure. We propose that the association of GKRP-P446L with multiple metabolic traits, including reduced T2D risk, can be attributed to its altered overall structure, which acts to hinder GK binding and nuclear import. This hypothesis is supported by the close approximation of Pro^446^ to Asp^413^ (as proposed by Choi et al. [47]) or Gln^443^ (as proposed by Beck et al. [49]), which appear to form a critical salt bridge with Arg^186^ of GK (Figures 1C and 1D).

### Disassembly of the GK–GKRP complex by glucose, F1P and GKA

Disassembly of the GK–GKRP complex was studied by TF using F1P, glucose and GKA with a GK/GKRP ratio of 3:1, as this ratio most closely mimics the ratio of these two proteins in the cytosol and provided optimal conditions to observe TF changes (see Figure 6). The concentration-dependency curves for these agents were hyperbolic for both GKRP-WT and GKRP-P446L (Tables 2–4 and Supplementary Figures S10–S12 at http://www.biochemj.org/bj/459/bj4590551add.htm). The glucose-dependency curve was monophasic for GKRP-WT, but biphasic for P446L-GKRP (Supplementary Figure S10). An apparent *K*_d_ value of 12 mM is consistent with the lower glucose *K*_d_ value of 5 mM for unbound GK-W99R/W167F/W257F under these conditions, and reflects the fact that GK-mediated glucose uptake and disposal by the intact hepatocyte is less efficient than expected based on the observed *S*_0.5_ value for purified GK [9]. The observation of two distinct glucose *K*_d_ values in the presence of GKRP-P446L (13.6 and 99.5 mM) is striking. This result is perhaps due to the altered ability of GKRP-P446L to bind glucose besides fructose phosphates (Supplementary Figure S7), which would result in increased, but separate, binding constants for the complex (one for GK and the other for GKRP). GKA was also highly effective in dissociating the GK–GKRP complex, with practically equal *K*_d_ values for GKRP-WT and GKRP-P446L (1.49 compared with 1.23 μM) (Supplementary Figure S11 and Table 4). F1P disrupted the 3:1 GK–GKRP complex very effectively (Supplementary Figure S12). However, the apparent *K*_d_ values for GKRP-WT and GKRP-P446L differed markedly (127 compared with 298 μM), resembling the 1:2 ratio of the ligand's binding constant for unbound GKRP (65 compared with 139 μM) (Figure 7). The lower affinity of F1P for GK-bound GKRP is likely the result of an altered phosphate ester binding site on GKRP when complexed with GK. We did not study the effect of F6P on complex assembly because the ΔTF value was too small (Figures 7C and 7D).

**Table 2:** D-Glucose binding by hGKRP (0.3 μM)–GK-W99R/W167F/W257F (1 μM) complexes at 20°C measured by TF of hGKRP Δ*F* is the relative fluorescence change at 340 nm.

Agents	Δ*F*	*K*_d_ (mM)	*H*	χ^2^
GKRP-WT and GK-W99R/W167F/W257F	−0.065	12	1	2.0×10^−5^
GKRP-P446L and GK-W99R/W167F/W257F	−0.064	13.6	1	1.0×10^−5^
GKRP-P446L and GK-W99R/W167F/W257F	−0.055	99.5	1	1.3×10^−5^

**Table 3:** F1P binding by hGKRP (0.3 μM)–GK-W99R/W167F/W257F (1 μM) at 20°C measured by TF of hGKRP Δ*F* is the relative fluorescence change at 340 nm.

Agents	Δ*F*	*K*_d_ (μM)	*H*	χ^2^
GKRP-WT and GK-W99R/W167F/W257F	−0.086	127	1	1.0×10^−5^
GKRP-P446L and GK-W99R/W167F/W257F	−0.095	298	1	1.0×10^−5^

**Table 4:** GKA binding by hGKRP (0.3 μM)–GK-W99R/W167F/W257F (1 μM) complexes at 20°C measured by TF of hGKRP Δ*F* is the relative fluorescence change at 340 nm.

Agents	Δ*F*	*K*_d_ (μM)	*H*	χ^2^
GKRP-WT and GK-W99R/W167F/W257F	−0.084	1.49	1	3.2×10^−5^
GKRP-P446L and GK-W99R/W167F/W257F	−0.065	1.23	1	1.0×10^−5^

### General discussion and outlook

#### The potential of TF for studying PPIs

The suitability of TF for structural and functional studies of PPIs depends on the number, location and functional involvement of native tryptophan residues [54]. The present study is the first to apply TF-based methodologies to investigate the GK–GKRP interaction. GK, when investigated in its monomeric form, is highly suitable for such studies due to the large nearly 2-fold increase in TF upon saturation with glucose, as well as the fact that mutation of all three of its native tryptophan residues can be performed without a loss of essential protein function [3,19,21,37,39,43,45,50,51,59,60]. TF studies with GKRP are more challenging because it has six tryptophan residues (Figure 1), one at the N-terminus that is associated with the Lid domain (Trp^19^), four in its SIS domains (Trp^222^, Trp^335^, Trp^461^ and Trp^484^), and one in the Lid itself (Trp^517^). The average quantum yield of GKRP is high, allowing for reliable studies at submicromolar protein concentrations, and the fluorescence signals are sufficient to allow for reliable functional and structural analyses. The ability to work with subnanomolar amounts compares favourably with alternative techniques such as crystallography, NMR or isothermal titration calorimetry, which require far more material. In the present study, we show that TF is capable of detecting even subtle conformational changes of GKRP in response to fructose-phosphate esters in agreement with the latest crystallography results [49]. The efficacy of this experimental approach is further demonstrated by its ability to detect qualitative and quantitative TF spectral differences between WT and mutant GKRPs, and to allow analysis of protein unfolding/refolding in the presence of urea. Most significant, this TF-based approach allowed a detailed description of the co-operative nature of GK–GKRP interactions. On the basis of the results already obtained with GK [21,43,50,51] it is anticipated that substitution of single tryptophan residues within GKRP will provide even greater insight into GKRP structure and function. However, the results obtained with GK-W99R/W167F/W257F must be viewed as model studies and their interpretation must be judged carefully because there are some differences compared with WT-GK, i.e. the *k*_cat_ value and Hill coefficient are reduced and the action of GKA, although highly effective in increasing glucose affinity, has been rendered glucose-independent ([51] and Supplementary Online Data).

The biological relevance of TF in the study of GK–GKRP interactions is also demonstrated upon comparison of our results with previously published data. Anderka et al. [19] characterized complex formation between hepatic GK and GKRP using isothermal titration calorimetry and SPR, and reported dissociation constants of 1–7 μM that are highly comparable with those found in the present study. Choi et al. [47], who used WT recombinant human GK and GKRP, reported *K*_d_ values for GK–GKRP complex formation via isothermal titration calorimetry that are consistent with those obtained in the present study. A relatively small contact area of about 2000 Å^2^ between the two proteins may be the structural prerequisite for a metabolic switch with relatively high association/dissociation rates [19,47,49].

#### On the biophysical basis of co-operative GK–GKRP interactions and their physiological significance

The observation that GK titration of GKRP results in sigmoidal concentration-dependency curves of increased TF, as well as the fact that this response is exquisitely sensitive to known physiological and pharmacological modifiers of complex assembly and disassembly, suggests a unique biochemical switch mechanism with important physiological implications (Figures 5–8 and Supplementary Figure S9). The shift in the GK dose–response curve to the right, combined with a large increase in the Hill coefficient in response to glucose, F1P and GKA, either alone or in combination, demonstrates that modulator molecules are particularly effective when cytosolic GK levels are low, i.e. when GK is retained in an inactive nuclear complex with GKRP. Modulation of this switch would result in corresponding equilibrium shifts between cytosolic and nuclear pools of GK. The *K*_d_ values of the small molecule modulators shown in the present paper are consistent with this interpretation (Tables 2–4 and Supplementary Figures S10–S12). We can only speculate about the biophysical basis of formation of this co-operative complex. The explanation may lie in the hysteretic behaviour of GK, as suggested by Bourbonais et al. [61], that GK release from GKRP in response to glucose is a slow process, exemplified by reaction progress curves with transition kinetics resembling those of activated or deactivated GK in response to glucose [3,17,41,45,51,61] (Supplementary Figure S13 at http://www.biochemj.org/bj/459/bj4590551add.htm).

#### The molecular basis of GK trafficking in hepatocytes during fasting and refeeding

The acute regulation of hepatic GK in response to feeding and fasting is poorly understood, partly due to the added complexity of altered subcellular compartmentalization of GK in response to these conditions and also because of the impact that the glucose-6-phosphatase system of the endoplasmic reticulum may have on glucose and glucose 6-phosphate levels and glucose uptake and release [7,62]. On the basis of the evidence that the cytosolic GKRP concentration appears to be much lower than that in the nucleus, but still appears to be essential for the interaction between GK and the NPC [1,2,10,11], we studied GK–GKRP interactions as they might occur in the cytosol at ratios ranging from 1:1 to 10:1 [1,2,9]. This approach demonstrated a sigmoidal GK dependency of complex formation, with Hill coefficients ranging from 1.3 to 3.2 depending on the experimental conditions. This suggests that GK binding to and release from GKRP functions as a co-operative switch that is activated by high levels of glucose, F1P, and pharmacological agents such as GKAs and GKRPIs, and is deactivated by low levels of these compounds. This critical step in glucose homoeostasis is thus analogous to that observed in the classical oxygen loading and releasing function of haemoglobin [63]. A similar co-operative switch in GK–GKRP assembly and disassembly can be postulated to exist in the nuclear space, which is readily accessible to low-molecular-mass ligands and consequently provides the molecular explanation for the compartmental reshuffling of GK during feeding–fasting transitions [1,2,8–11]. This subcellular redistribution of GK implies the rapid movement of significant amounts of protein and considerable expenditure of metabolic energy. A dramatic change in the equilibrium of the GK–GKRP complex must occur to allow for the timely and efficient subcellular redistribution of GK in this manner.

#### Pathophysiological and pharmacological significance of the hepatic GK–GKRP switch

Activation of the GK–GKRP switch by F1P is not only of physiological significance, but could also be instrumental in the metabolic dysregulation that results from excessive dietary fructose intake, which is hypothesized to be a major contributor to metabolic diseases such as obesity and T2D [64–66]. Fructose levels of 1 mM or more in the portal vein would result in a high degree of F1P production by hepatic fructokinase C, greatly enhancing GK mobilization and activation. This hyperactivation of GK would result in increased glycolysis, glycogen synthesis and *de novo* lipogenesis, all of which may contribute to increased insulin resistance, obesity, hepatosteatosis and T2D [67]. Excessive activation of the GK–GKRP switch by F1P may also amplify other cellular mechanisms (such as ATP depletion and uric acid overproduction) that could contribute to disease progression [65]. In contrast, low amounts of fructose appear to have a beneficial effect on hepatic glucose metabolism [68,69], rendering the GK–GKRP switch optimal in its ability to respond to common carbohydrate meals that contain both sucrose and starches. In the context of GKAs, a recent report of a Phase II trial of subjects with T2D treated with the GKA MK0941 observed that a low dose of the drug resulted in a slow-onset long-lasting beneficial effect, whereas higher doses of the drug resulted in fast-onset transient effects associated with an increase in blood lipids and cardiovascular side effects [34]. Extrapolating from these clinical results with a GKA indicates that similar difficulties might be encountered with the newly discovered GKRPIs when applied to treat human disease [22].

#### Contribution to our understanding of the molecular mechanisms explaining genetic associations of the GKRP-P449L variant with metabolic traits and diabetes risk

**Figure 9:**
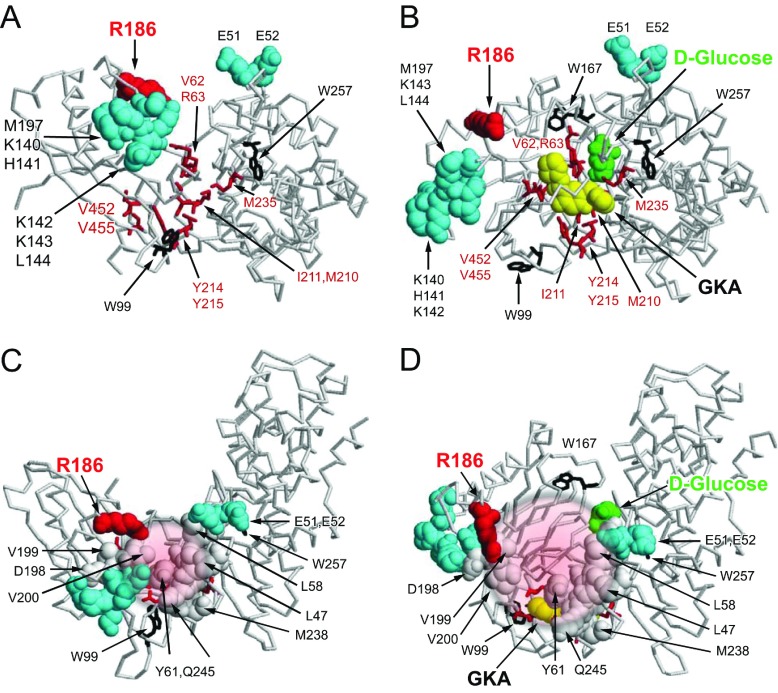
Effects of glucose, fructose-phosphate esters, GKAs and GKRPIs on the GK–GKRP complex The Figure summarizes the structural information on the GK–GKRP complex under the influence of glucose and known allosteric ligands of the two participating proteins ([21,22,38,47–49] and the present study). (**A** and **B**) The results of mutational analyses [21]. (**C** and **D**) The combined results of mutational [21] and crystallographic [22,47–49] analyses of these interactions [note that the orientation differs from that of (**A**) and (**B**) (an approximately 90° rotation towards the face of the page) in order to emphasize that the site of GK–GKRP interaction is distinct from that of GK–GKA]. All structural information is projected on to the GK structures published by Kamata et al. [38], and the tryptophan residues are depicted as black stick structures for improved orientation. (**A**) The allosteric modifier region of GK with two distinct binding sites for GKRP and GKA in the open conformation. Amino acids that affect interaction with GKRP are shown as space-filled structures in cyan. Arg^186^ of GK is presented in magenta [47,49] to show its close vicinity to other amino acids in the small lobe of GK that have been implicated in GKRP binding. GKA-contacting amino acids are shown as red stick models. Activation of GK by glucose (green) combined with a GKA (yellow) profoundly alters the proposed GKRP-binding site by affecting groups of amino acids involved in binding including the critical Trp^99^ for binding (**B**). (**C** and **D**) The allosteric binding site for GKRP is indicated in grey space-filled structures for eight of the 15 identified contact amino acids [47,49]. Amino acids immediately surrounding the GKRP contact site are those identified by mutational analysis to influence GKRP binding to GK and are presented as space-filled structures in cyan (see also **A** and **B**) [21]. The GK-binding site for GKRP is drawn schematically as a small circular patch in (**C**) to delineate where the two proteins interact in the complex and a larger patch in (**D**) to delineate the conformational change that dislodges the inhibitor when glucose and/or GKA are bound to GK.

Kinetic studies of GKRP-P446L have demonstrated a reduced ability of this variant protein to inhibit GK as a consequence of reduced responsiveness to F6P, and cellular studies have shown impaired GK binding and nuclear storage [28,30,31]. Subsequent alterations in GK localization and activity may explain the association of this GKRP variant with metabolic traits such as fasting glucose and triacylglycerol levels [33]. The present study expands the characterization of GKRP-P446L. First, we show that structural instability or impaired protein folding are unlikely to be major contributors to GKRP-P446L dysfunction (Figure 4). Secondly, the increase in the GK *S*_0.5_ value for binding to GKRP-P446L, the reduced affinity of GK for GKRP-P446L in the presence of high glucose and GKA, the abnormal constancy of TF maxima under various conditions, and, finally, the reduced co-operativity with GK, suggest a narrower conformational range for this variant protein (Figures 5–8 and Table 1, and Supplementary Figure S9). There is also evidence for reduced fructose-phosphate ester binding by GKRP-P446L (Supplementary Figure S12). Taken together, these data indicate significant conformational differences as a result of the P446L substitution, which could limit the ability of GKRP to facilitate nuclear uptake of GK via the NPC and act as an effective storage partner in the nucleus. The recently disclosed crystal structure of human GKRP and the binary xGK–xGKRP and hGK–rGKRP complex [47–49] suggests that Pro^446^ is located in one of the surface loops of the SIS-2 domain, a region with high surface entropy, that may explain both the conformational and functional alterations observed in the present study (Figures 1 and 9). Proline is a helix-disrupting amino acid [70], and replacing it with leucine may destabilize this region.

In Figure 9, the results of our previous mutational analyses of the GK–GKRP interaction [21] are integrated with recent crystallographic information [47–49]. This clearly shows that the SIS-2 region is involved directly in GKRP binding to GK [47,49]. The GKRP-binding site is located between two groups of amino acids, composed of Glu^51^ and Glu^52^ in the large lobe and Lys^140^–Leu^144^ and Met^197^ in the small lobe. Both patches are clearly separate to the GKA-binding site (Figure 9A). Activation of GK by glucose or GKAs results in a large spatial separation of these two groups which would dislodge GKRP (Figure 9B). Figures 9(C) and 9(D) further illustrate how the large conformational change in GK in response to glucose is the main driving force behind disruption of the inactive GK–GKRP complex. The position of Pro^446^ on the surface of GKRP and its close vicinity to Asp^413^ [47] and Gln^443^ [49], which appear to form a salt bridge with Arg^186^ of GK, is almost certainly of functional significance, and the P446L substitution may thus interfere directly with binding to GK (compare with Figure 1D). The widely dispersed intramolecular locations of 16 other rare GKRP variants associated with serum triacylglycerol levels, and their spatial relationship to the allosteric fructose phosphate-, GKRPI- and GK-binding sites, suggests that the molecular mechanisms that underpin these mutants differ greatly (Figure 1C). Further pursuit of TF in the study of rare genetic variants in GKRP promises to be useful in understanding these distinct molecular mechanisms in greater depth.

#### Outlook

The results of the present study suggest that TF is an excellent means to improve our understanding of GKRP structure and function and its interaction with GK. Exploration of the co-operative nature of the GK–GKRP interaction using rapid mixing studies may better resolve the apparent slow transition of GKRP binding to and dissociation from GK. Such studies may also be extended to other disease-associated GKRP variants found in humans. Also, utilization of our advanced biophysical knowledge of the GK–GKRP interaction as a co-operative metabolite- and drug-responsive switch will deepen our understanding of the physiological chemistry and pharmacology of hepatic glucose metabolism and its contribution to disease pathology.

## Online data

Supplementary data
