# Supplementary material for: Analysis of the co-operative interaction between the allosterically regulated proteins GK and GKRP using tryptophan fluorescence
Source: Biochem J. 2014 Apr 11;459(Pt 3):551–64. doi: 10.1042/BJ20131363 (PMC4109836; doi:10.1042/BJ20131363)
Supplement: Supplementary data [file bj4590551add.pdf]

BJ Biomolecules

Biochem. J. (2014) **459**, 551–564 (Printed in Great Britain) doi:10.1042/BJ20131363

online data

SUPPLEMENTARY ONLINE DATA

Analysis of the co-operative interaction between the allosterically regulated proteins GK and GKRP using tryptophan fluorescence

Bogumil ZELENT*, Anne RAIMONDO†, Amy BARRETT†, Carol W. BUETTGER*, Pan CHEN*, Anna L. GLOYN† and Franz M. MATSCHINSKY*[1]

*Department of Biochemistry and Biophysics and Institute for Diabetes, Obesity and Metabolism, Perelman School of Medicine, University of Pennsylvania, Philadelphia, PA, U.S.A.
†Oxford Centre for Diabetes Endocrinology & Metabolism, University of Oxford, Oxford, U.K.

RESULTS AND DISCUSSION

Co-operativity and allostery in GK–GKRP complex formation: conceptual and terminological considerations

The present study describes experiments which demonstrate that the concentration-dependency curve characterizing binding of GK by GKRP is sigmoidal deviating from a classical hyperbolic binding isotherm. TF of GKRP, which contains six tryptophan residues distributed throughout the molecule, was used as a sensitive indicator of this interaction with a non-fluorescent GK reagent (Figure S1A). Glucose, F1P, a drug that activates GK (a GKA) and various combinations of these magnified this sigmoidicity manifold resulting in very steep effector-dependency curves manifesting switch characteristics (Figures S5–S8). This phenomenology is strikingly illustrated by the summary of the results in linearized plots using the Hill equation (Supplementary Figure S9). Such phenomenology is commonly characterized by the concepts of co-operativity and allostery, best exemplified by oxygen binding to haemoglobin. The classical models of Monod–Wyman–Changeux (the MWC selection or symmetry model [1]) and of Koshland–Nemethy–Filmer (the KNF instruction or sequential model [2]) are commonly used to provide mechanistic explanations for co-operativity and allostery. An essential common postulate of these two models is the oligomeric nature of the proteins exhibiting co-operativity. Allostery, the other common feature of these models (a term derived from the Greek and meaning 'of different shape'), refers to the fact that proteins may be regulated by molecules that differ structurally from the substrate of an enzyme or target of a regulatory protein implying also the existence of a separate binding site [3].

The method of choice would be to apply approved concepts of co-operativity and allostery (the MWC and KNF models) for understanding at the molecular level the sigmoidicity of the concentration-dependency curve for assembly of the GK–GKRP complex, but this presents difficulties because of the unique biochemistry and monomeric nature of the two binding partners of the complex. The catalytic function of GK clearly shows co-operativity and allostery. The concentration-dependency curve for glucose binding to GK is, however, hyperbolic as expression of a single binding site of the monomeric enzyme, whereas that for phosphorylation of glucose by GK in the presence of Mg-ATP is sigmoidal/co-operative [4], which is explained kinetically by two related models termed the LIST (ligand-induced slow transition) [5,6] and the mnemonic [7,8] models respectively, both of which are based on the hysteretic nature of the protein [7,8]; hysteresis referring to the fact that the ligand-induced transition from low-affinity to high-affinity conformations of the protein is slow compared with its k_{cat} value [5,7,9]. GK has a well-defined allosteric modifier region remote from the active site with two distinct receptors for GKA and GKRP, one activating the other inhibiting the enzyme [10,11]. An additional allosteric site was recently identified that binds the proapoptotic factor BAD which increases the V_{max} value when occupied [12]. GKRP also clearly shows co-operativity and allostery. The 'active site' of GKRP mediates binding to its receptor on GK. The binding curve is sigmoidal/co-operative as described in the present paper and by Choi et al. [13]. GKRP has two separate allosteric sites, one binding fructose-phosphate esters [13,14] which either enhance (fructose 6-phosphate) or inhibit (fructose 1-phosphate) binding to GK (to result in opposite TF changes of GKRP; Figure S7) and the other binding a pharmacological disruptor of the GK–GKRP complex [15], in the present paper referred to as GKRPIs and discussed in the legend of Figure S1.

The Hill equation, first published 100 years ago as a purely descriptive formula to characterize co-operativity of oxygen binding to haemoglobin [16,17], allows us to describe similarly the co-operative interaction of the GK and GKRP monomers as influenced by glucose (binding to the substrate site of GK) and various allosteric effector molecules (fructose-phosphate esters or GKRPIs and GKAs binding to allosteric sites of GKRP and GK respectively). However, a mechanistic explanation for this phenomenology, potentially of high biological significance, remains to be found.

Further critical experimental material and conceptual commentary on the biochemical characteristics of GK-W99R/W167F/W257F

To facilitate interpretation of our results we characterized GK-W99R/W167F/W257F in greater detail. We have already published [4] the following biochemical characteristics of the tryptophan-free GK reagent (mutant compared with WT enzyme): recombinant protein yield = 19.2 compared with 43.5 mg/l; $k_{cat} = 6.14$ compared with 62.8 1/s; glucose $S_{0.5} = 4.8$ compared with 7.5 mM; H (glucose) = 1.45 compared with 1.77; ATP $K_m = 1.96$ compared with 0.49; and relative activity index $GI = 0.33$ compared with 1.0 as well as normal responsiveness to GKRP inhibition and GKA activation. The 90 % reduction in the k_{cat} value is most noteworthy, yet is probably least significant for the present binding studies. However, the near total glucose dependency of GKA action seen with GK-WT is lost in Trp^{99}-modified GK and is highly relevant (Figure S2 and Tables S2 and S3). Although binding to GK-WT in the absence of glucose was barely detectable, affinity was near maximal at a glucose concentration of 7.5 mM ($K_d = 0.41$ μM; Figure S2). It is remarkable that the affinity of the known Trp^{99} GK mutants (GK-W99C, GK-W99L and GK-W99R) and GK-W99R/W257F

[1] To whom correspondence should be addressed (email matsch@mail.med.upenn.edu).

© 2014 The Author(s)
This is an Open Access article distributed under the terms of the Creative Commons Attribution Licence (CC-BY) (http://creativecommons.org/licenses/by/3.0/) which permits unrestricted use, distribution and reproduction in any medium, provided the original work is properly cited.

Biochemical Journal www.biochemj.org

have highly comparable glucose-independent K_d values for GKA (Table S2). To explore the allosteric site of non-fluorescent GK-W99R/W167F/W257F in greater detail, we introduced an ectopic tryptophan residue at position Thr[65]. The loop spanning residues Val[62] to Gly[72] undergoes a drastic rearrangement during GK activation by sugars or GKA [10,11]. We therefore expected that the TF signal for GK-T65W/W99R/W167F/W257F would allow us to determine the K_d value of this protein for GKA. Ultimately, its affinity for GKA was indeed comparable with that of GK-WT ($K_d = 0.51\ \mu$M; Figure S3). At saturating GKA levels, glucose affinity was increased approximately 6-fold, which is comparable with the degree of activation observed for GK-WT (Figure S3). These data clearly demonstrate that the process of allosteric activation remains intact for GK-T65W/W99R/W167F/W257F (Figures S3 and S4, and Table S3). The observed lowering of the Hill coefficient of GK-W99R/W167F/W257F can be explained by the reduced k_{cat} value of the modified GK [7,8]. Time-dependency experiments allowed us to calculate the activation energy (E_a) of ligand-induced slow conformational transitions for WT and mutant GK proteins (K140E, S263P, M298K, S336L, W99R and W99R/W257F) (Figure S1 and Table S1). Ligand binding resulted in a slow transition from a low to a high sugar affinity conformation of the enzyme, a response classically seen with hysteretic enzymes [5–9]. GKA activation of GK-K140E, GK-S263P, GK-M298K and GK-S336L required the presence of MH, whereas GK-W99R and GK-W99R/W257F (here referred to as the W167 enzyme) responded to GKA alone (Figure S4). The rate constants of GK-W99R and GK-W99R/W257F were highest (Table S1), which seems to indicate that Trp[99] sterically hinders GKA access in the open conformation of the WT enzyme (Figures S9A and S9B). The rate of transition was increased by temperature as indicated by the transition times and rate constants. The calculated E_a value for ligand-induced activation ranged from 48.8 to 73.1 kJ/mol and was practically the same for GK-WT, GK-W99R and GK-W99R/W257F. These data demonstrate that neither Trp[99] substitution nor drastic reengineering of the GK protein compromise the basic process of ligand-induced GK activation. Table S3 shows a range of GKA K_d values for all Trp[99] substitution mutants varying from 0.4 to 0.51 μM, suggesting that the GKA affinity for GK when complexed with GKRP was markedly decreased compared with that of free GK, similar to the effect of complex formation on glucose binding to GK and on F1P binding to GKRP (Tables S1–S4 and Figures S10–S12 and the glucose affinity of 4.8 mM for GK-W99R/W167F/W257F described above). Because of the described quantitative and qualitative impact that the mutational modification exerts on the GK reagent of our assay system we describe and interpret the present experiments as model studies, but conclude that extrapolation to the normal situation are highly meaningful and relevant.

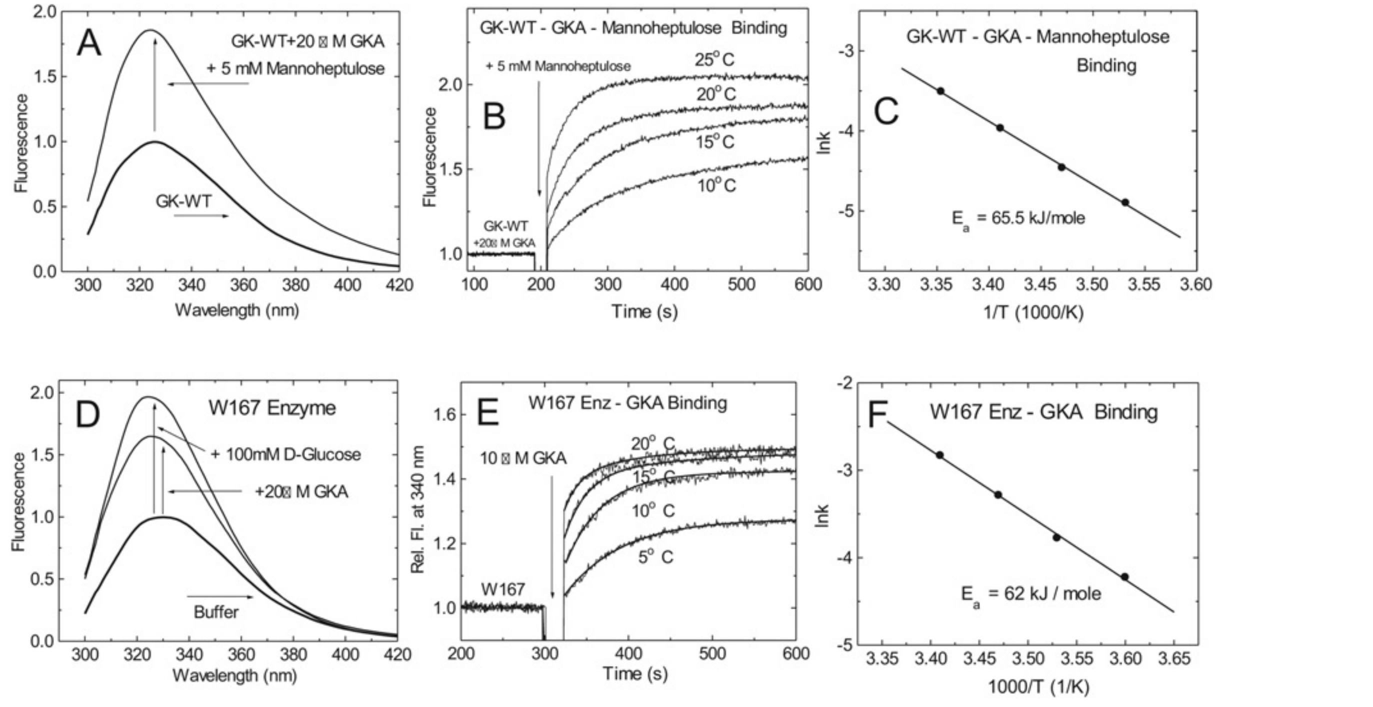

Figure S1 TF measurements illustrating the slow ligand-induced conformational transition of GK-WT and GK-W99R/W257F (W167 enzyme)

(**A**) TF of ~1 μM GK-WT in the presence or absence of 5 mM MH plus 20 μM GKA. Note that the K_d values for MH decreased from 17 mM to 1 mM in the presence of GKA. (**B**) Temperature dependency of the MH/GKA-induced transition process. Binding of GKA requires the presence of both MH and GKA. (**C**) Arrhenius plot of the data in (**B**) allowing for calculation of the activation energy of the transition process. (**D**) TF of the W167 enzyme (i.e. GK-W99R/W257F) in the presence of 20 μM GKA or 100 mM D-glucose. (**E**) Kinetics of the temperature-dependent increase in TF following the addition of 10 μM GKA. For the W167 enzyme, the increase in TF in the presence of GKA is sugar-independent. (**F**) Arrhenius plot of GKA-induced activation of the W167 enzyme. (**A**, **B** and **C**) are modified from [18]: American Diabetes Association, Glucokinase Activators for Diabetes Therapy: May 2010 status report, American Diabetes Association, 2011. Copyright and all rights reserved. Material from this publication has been used with the permission of American Diabetes Association. © 2011, American Diabetes Association.

© 2014 The Author(s)
This is an Open Access article distributed under the terms of the Creative Commons Attribution Licence (CC-BY) (http://creativecommons.org/licenses/by/3.0/) which permits unrestricted use, distribution and reproduction in any medium, provided the original work is properly cited.

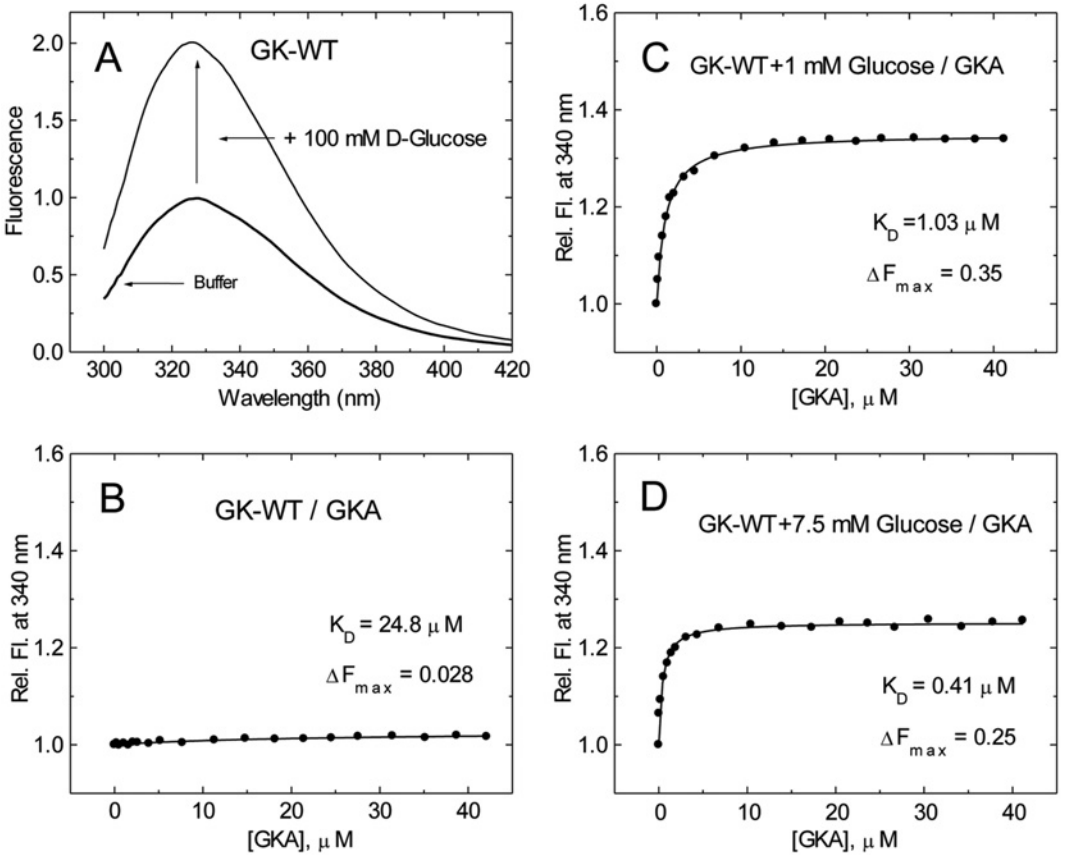

Figure S2 Relative change in TF for 0.9 μM GK-WT at 20°C

In the presence or absence of 100 mM D-glucose (**A**), in the presence of increasing amounts of GKA (**B**), in the presence of 1 mM D-glucose and increasing amounts of GKA (**C**), and in the presence of 7.5 mM D-glucose and increasing amounts of GKA ($\lambda_{exc} = 295$ nm; $\lambda_{em} = 340$ nm).

© 2014 The Author(s)
This is an Open Access article distributed under the terms of the Creative Commons Attribution Licence (CC-BY) (http://creativecommons.org/licenses/by/3.0/) which permits unrestricted use, distribution and reproduction in any medium, provided the original work is properly cited.

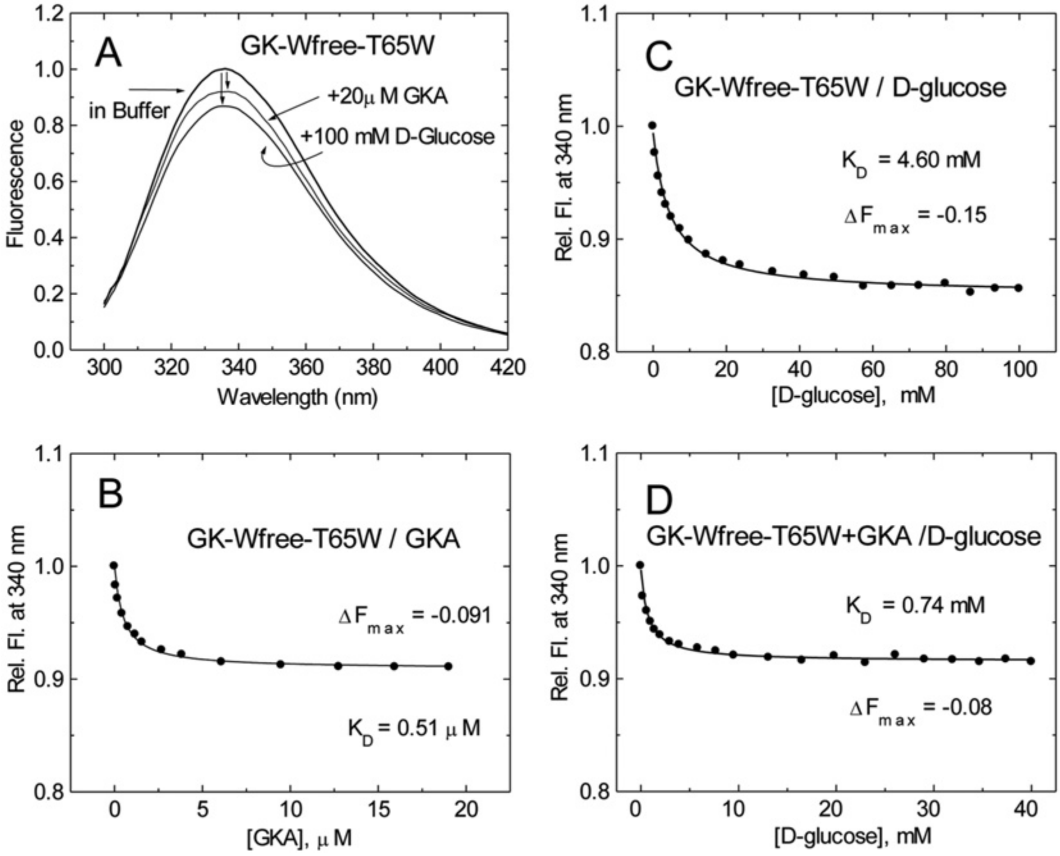

Figure S3 Fluorimetric evidence for glucose binding and glucose-independent GKA binding by GK-T65W/W99R/W167F/W257F at 25 °C

(**A**) Decrease in TF in the presence of 20 μM GKA or in the presence of 100 mM D-glucose. (**B**) Effect of increasing amounts of GKA on this decrease. (**C**) Decrease in TF upon titration with D-glucose and (**D**) decrease in TF upon titration with D-glucose in the presence of 20 μM GKA ($\lambda_{exc} = 295$ nm; $\lambda_{em} = 340$ nm).

© 2014 The Author(s)
This is an Open Access article distributed under the terms of the Creative Commons Attribution Licence (CC-BY) (http://creativecommons.org/licenses/by/3.0/) which permits unrestricted use, distribution and reproduction in any medium, provided the original work is properly cited.

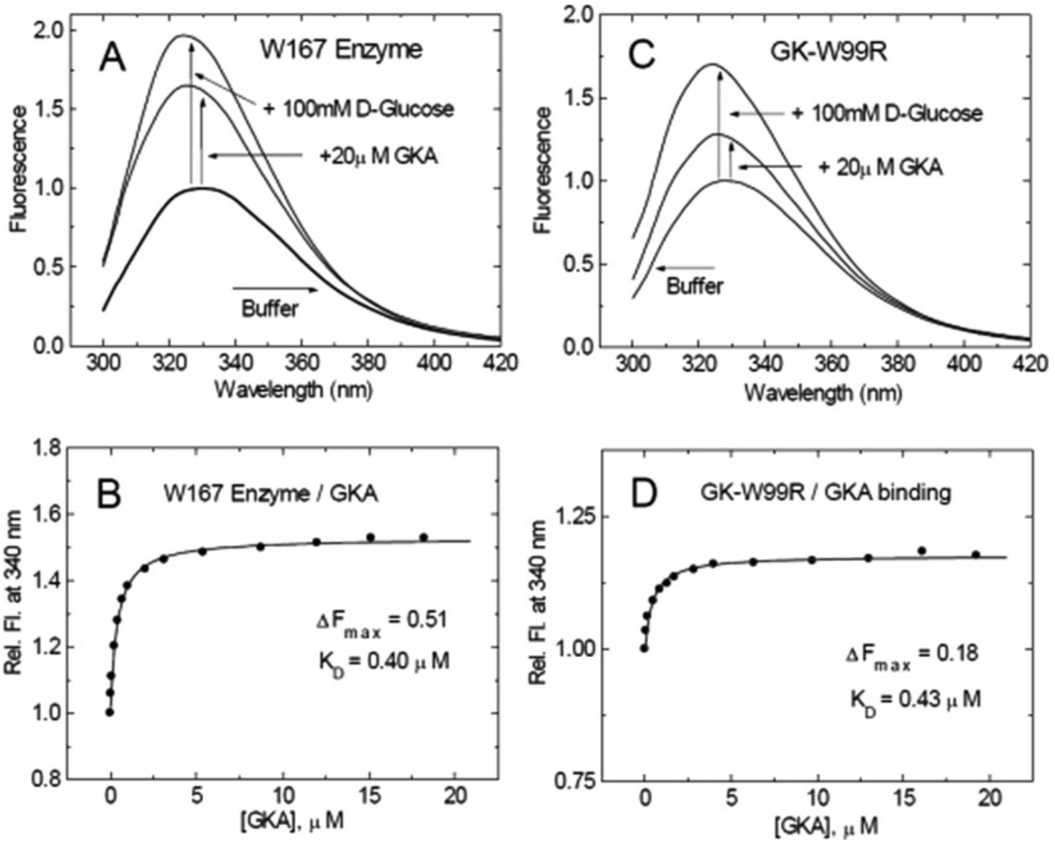

Figure S4 Fluorimetric evidence for glucose-independent GKA binding by the W167 enzyme (i.e. GK-W99R/W257F) and GK-W99R

(**A** and **C**) Increase in the presence of 20 μM GKA or 100 mM D-glucose. (**B** and **D**) Effect of increasing amounts of GKA on this increase ($\lambda_{exc} = 295$ nm; $\lambda_{em} = 340$ nm).

© 2014 The Author(s)
This is an Open Access article distributed under the terms of the Creative Commons Attribution Licence (CC-BY) (http://creativecommons.org/licenses/by/3.0/) which permits unrestricted use, distribution and reproduction in any medium, provided the original work is properly cited.

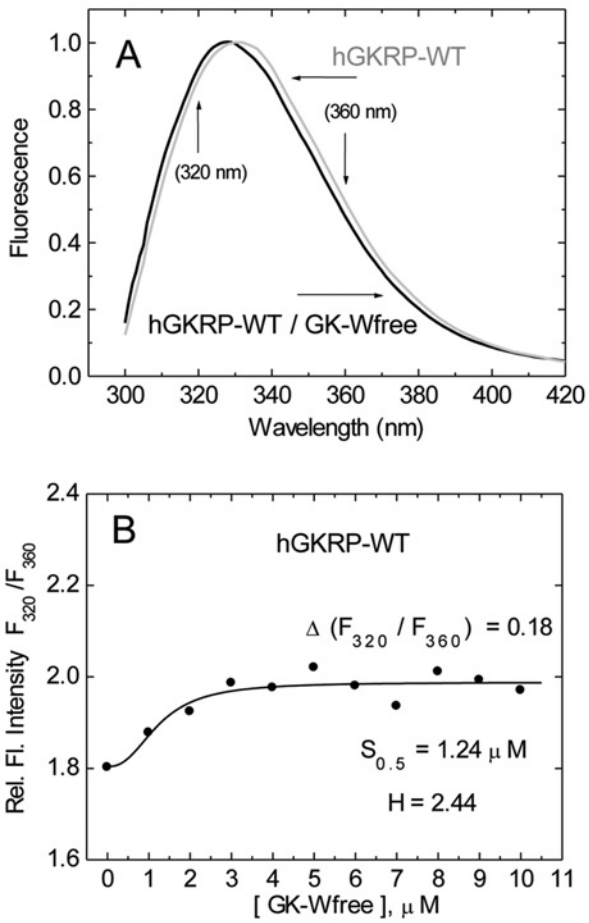

Figure S5 Demonstration of the GK-induced fluorescence blue shift of GKRP

(**A**) Normalized TF spectra for 0.3 μM GKRP-WT showing a 5 nm blue shift in the presence of 5 μM GK-W99R/W167F/W257F ($\lambda_{exc} = 295$ nm). (**B**) Relative fluorescence intensity increase at 320 and 360 nm (F_{320}/F_{360}) as a function of increasing amount of GK-W99R/W167F/W257F.

© 2014 The Author(s)
This is an Open Access article distributed under the terms of the Creative Commons Attribution Licence (CC-BY) (http://creativecommons.org/licenses/by/3.0/) which permits unrestricted use, distribution and reproduction in any medium, provided the original work is properly cited.

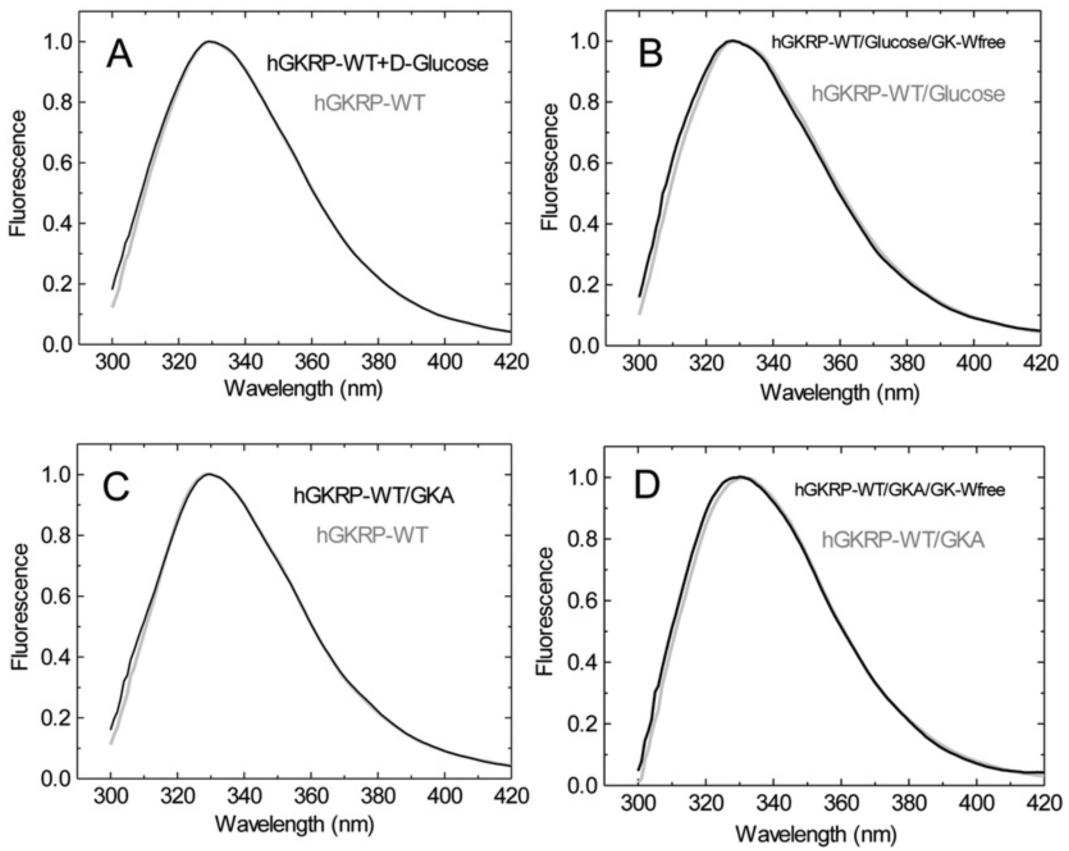

Figure S6 Normalized TF spectra for 0.3 μM GKRP-WT

(**A**) Normalized TF spectra in the presence or absence of 100 mM D-glucose. (**B**) The effect of 5 μM GK-W99R/W167F/W257F on the TF of GKRP-WT in the presence of 100 mM D-glucose. (**C**) Normalized TF spectra in the presence or absence of 20 μM GKA. (**D**) The effect of 5 μM GK-W99R/W167F/W257F on the TF spectrum of GKRP-WT in the presence of 20 μM GKA ($\lambda_{exc} = 295$ nm).

© 2014 The Author(s)
This is an Open Access article distributed under the terms of the Creative Commons Attribution Licence (CC-BY) (http://creativecommons.org/licenses/by/3.0/) which permits unrestricted use, distribution and reproduction in any medium, provided the original work is properly cited.

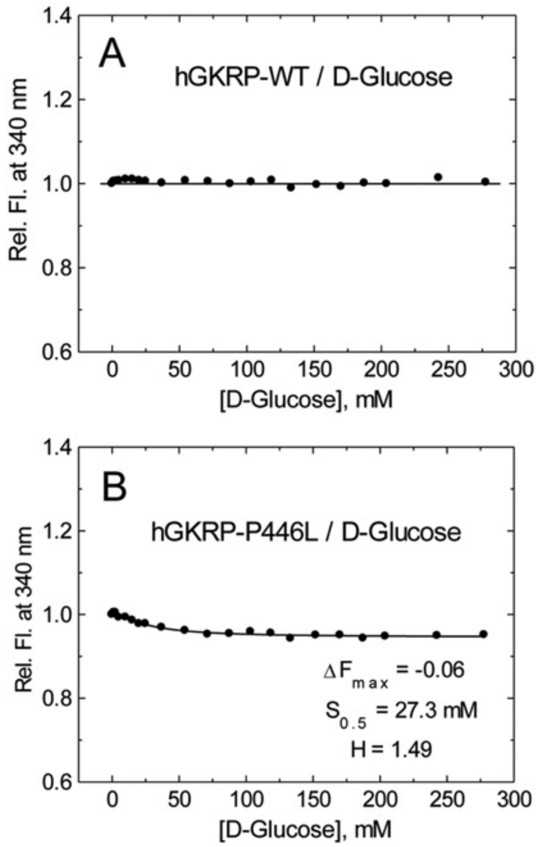

Figure S7 Relative change in TF for 0.3 μM GKRP in the presence of increasing amounts of D-glucose

For GKRP-WT (**A**) and for GKRP-P446L (**B**) ($\lambda_{exc} = 295$ nm; $\lambda_{em} = 340$ nm).

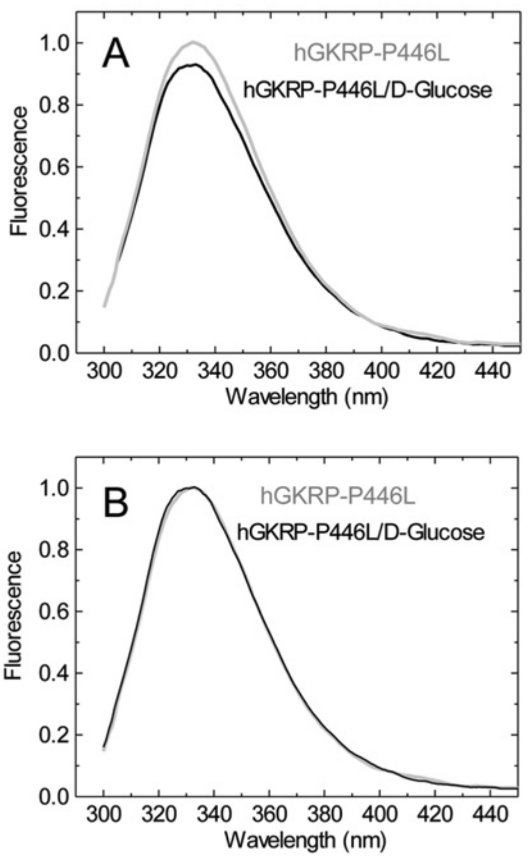

Figure S8 Atypical glucose binding to GKRP-P446L

(**A**) TF spectra for 0.3 μM GKRP-P446L in the presence or absence of 100 mM D-glucose. (**B**) Normalized TF of GKRP-P446L from (**A**).

© 2014 The Author(s)
This is an Open Access article distributed under the terms of the Creative Commons Attribution Licence (CC-BY) (http://creativecommons.org/licenses/by/3.0/) which permits unrestricted use, distribution and reproduction in any medium, provided the original work is properly cited.

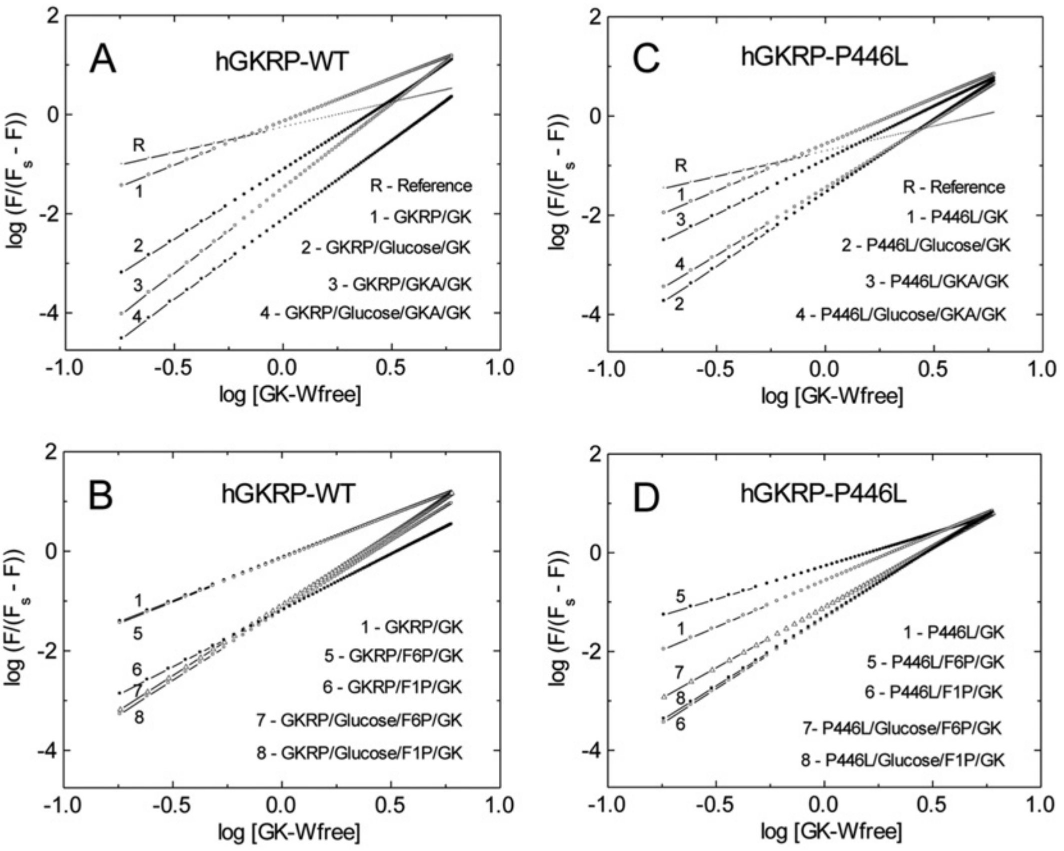

Figure S9 Co-operativity of GK–GKRP complex assembly presented as Hill plots

Hill plots for GK-W99R/W167F/W257F binding to GKRP-WT and GKRP-P446L as measured by TF GKRP fluorescence as a function of glucose and GKA binding (**A** and **C**) and as a function of F6P, F1P and glucose binding as indicated (**B** and **D**). The plots are based on data shown in Figures S6–S8.

© 2014 The Author(s)
This is an Open Access article distributed under the terms of the Creative Commons Attribution Licence (CC-BY) (http://creativecommons.org/licenses/by/3.0/) which permits unrestricted use, distribution and reproduction in any medium, provided the original work is properly cited.

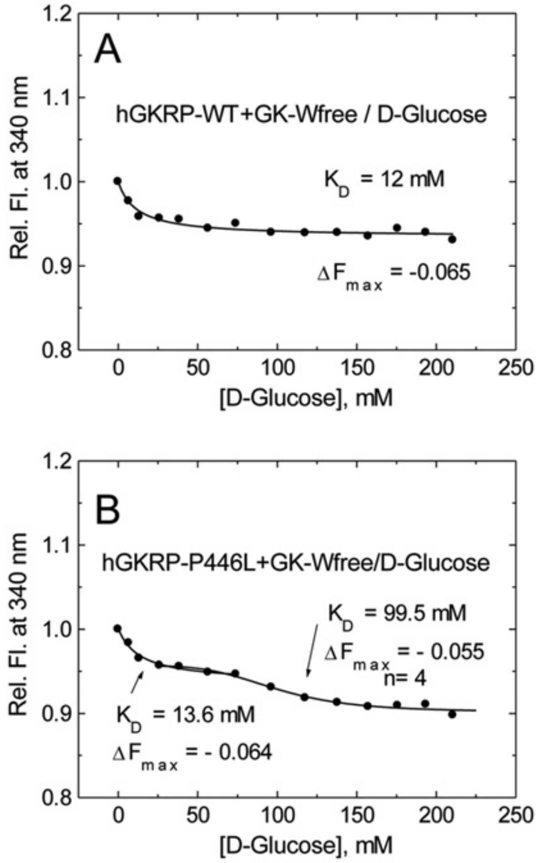

Figure S10 Relative change in TF for 0.3 μM GKRP and 1 μM GK in the presence of increasing amounts of D-glucose

For GKRP-WT (**A**) and GKRP-P446L (**B**) ($\lambda_{exc} = 295$ nm; $\lambda_{em} = 340$ nm). GK-Wfree, GK-W99R/W167F/W257F.

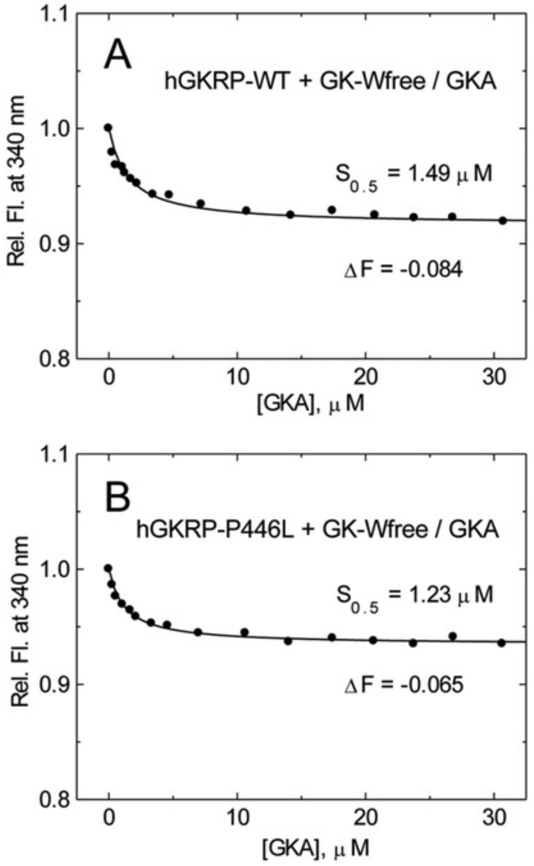

Figure S11 Relative decrease in TF for 0.3 μM GKRP bound to GK (1 μM) in the presence of increasing amounts of GKA

For GKRP-WT (**A**) and for GKRP-P446L (**B**) ($\lambda_{exc} = 295$ nm; $\lambda_{em} = 340$ nm). GK-Wfree, GK-W99R/W167F/W257F.

© 2014 The Author(s)
This is an Open Access article distributed under the terms of the Creative Commons Attribution Licence (CC-BY) (http://creativecommons.org/licenses/by/3.0/) which permits unrestricted use, distribution and reproduction in any medium, provided the original work is properly cited.

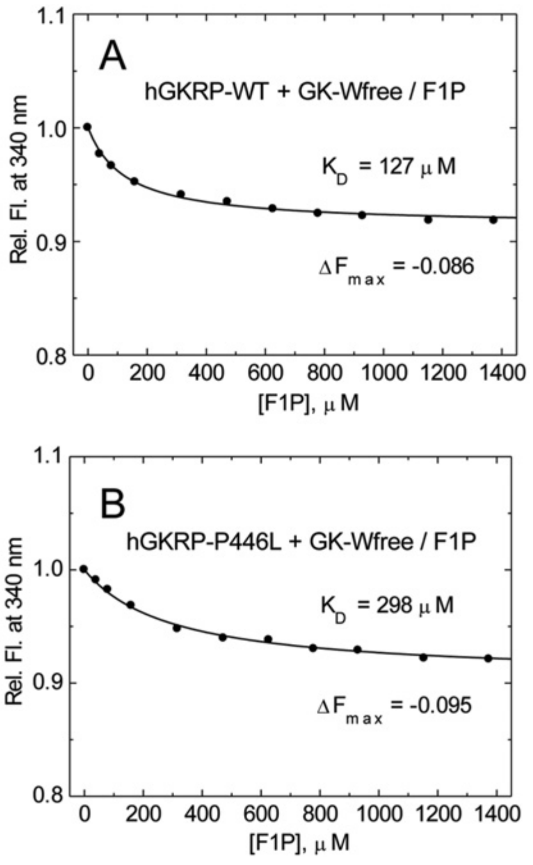

Figure S12 Relative decrease in TF for 0.3 μM GKRP bound to 1 μM GK-W99R/W167F/W257F in the presence of increasing amounts of F1P

For GKRP-WT (**A**) and GKRP-P446L (**B**) ($\lambda_{exc} = 295$ nm; $\lambda_{em} = 340$ nm).

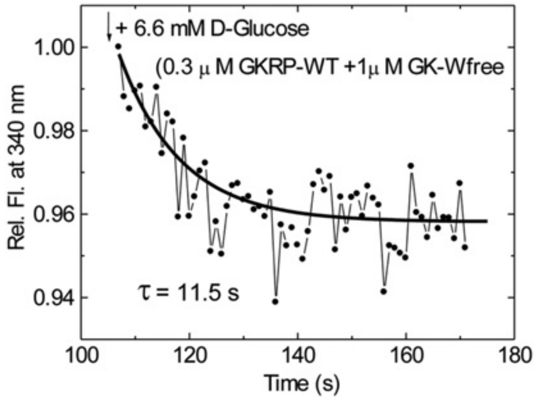

Figure S13 Time-dependent decrease in GKRP-WT TF upon addition of 6.6 mM D-glucose containing 0.3 μM GKRP and 1 μM GK in buffer (pH 7.2)

Data fitted to a mono ExpDecay function yields values of $\tau = 11$ s and $A = 0.04$ ($\lambda_{exc} = 295$ nm; $\lambda_{em} = 340$ nm). GK-Wfree, GK-W99R/W167F/W257F.

© 2014 The Author(s)
This is an Open Access article distributed under the terms of the Creative Commons Attribution Licence (CC-BY) (http://creativecommons.org/licenses/by/3.0/) which permits unrestricted use, distribution and reproduction in any medium, provided the original work is properly cited.

Table S1 Temperature dependence of the kinetics for ligand binding to GK-WT and GK mutants measured by TF

Results are the thermodynamic parameters for the activation process.

GK	Ligand(s) (concentration)	Temperature (°C)	Time (s)	k (s^{-1})	ΔE_a (kJ/mol)	$\Delta H°$ (kJ/mol)	$\Delta S°$ (kJ/molK)	$\Delta G°$ (kJ/mol)
GK-WT	GKA (20 μM)/MH (1 mM)	25.0	33.4	2.99×10^{-2}	65.5	63.0	− 62.8	81.7
		20.0	52.9	1.89×10^{-2}				
		15.0	86.6	1.15×10^{-2}				
		10.0	134.4	0.74×10^{-2}				
GK-S336L	GKA (20 μM)/MH (10 mM)	25.4	37	2.70×10^{-2}	48.8	44.3	− 119.5	81.9
		20.1	52.7	1.90×10^{-2}				
		15.0	72.2	1.39×10^{-2}				
		10.1	106.3	0.94×10^{-2}				
GK-M298K	GKA (20 μM)/MH (10 mM)	25.4	34.9	2.87×10^{-2}	60.7	58.2	− 79.2	81.8
		20.1	54.1	1.85×10^{-2}				
		15.0	85.2	1.17×10^{-2}				
		10.1	126.9	0.79×10^{-2}				
GK-S263P	GKA (20 μM)/MH (10 mM)	25.4	32.7	3.06×10^{-2}	53.6	51.1	− 99.9	80.9
		20.1	47.6	2.10×10^{-2}				
		15.0	64.0	1.56×10^{-2}				
		10.1	105.7	0.95×10^{-2}				
GK-K140E	GKA (20 μM)/MH (10 mM)	25.4	20.6	4.85×10^{-2}	73.1	70.6	− 33.7	80.6
		20.1	38.3	2.61×10^{-2}				
		15.0	58.5	1.71×10^{-2}				
		10.1	101.7	0.98×10^{-2}				
GK-W99R	GKA (20 μM)	20.1	11.8	8.48×10^{-2}	60.7	58.2	− 66.8	78.1
		15.0	19.6	5.38×10^{-2}				
		10.1	30.1	3.32×10^{-2}				
		4.6	44.7	2.24×10^{-2}				
GK-W99R/W257F	GKA (20 μM)	20.1	17.0	5.88×10^{-2}	61.5	59.0	− 67.2	79.0
		15.0	26.8	3.73×10^{-2}				
		10.1	43.7	2.29×10^{-2}				
		4.6	68.5	1.46×10^{-2}				

Table S2 GKA binding by GK-WT at 20°C in the absence and presence of D-glucose measured by TF

All reactions used 0.9 μM GK-WT. ΔF is the relative fluorescence change at 340 nm.

D-glucose (mM)	ΔF	K_d (μM)	H	χ^2
None	0.028	25	1	1.0×10^{-5}
1	0.35	1.03	1	4.4×10^{-5}
7.5	0.25	0.41	1	23×10^{-5}

Table S3 Glucose and GKA binding by GK-W99R/W257F, GK-W99R and GK-T65W/W99R/W167F/W257F measured at 20°C by TF of hGKRP and GK mutants

ΔF is the relative fluorescence change at 340 nm.

Combinations of proteins and ligands	ΔF	K_d (μM)	H	χ^2
GK-W99R/W257F	0.51	0.40	1	9.7×10^{-5}
GK-W99R	0.18	0.43	1	5.8×10^{-5}
GK-T65W/W99R/W167F/W257F	− 0.091	0.51	1	1.0×10^{-5}
GK-T65W/W99R/W167F/W257F	− 0.15	4.60*	1	1.2×10^{-5}
GK-T65W/W99R/W167W257F and GKA	− 0.08	0.74*	1	1.0×10^{-5}

*D-glucose binding at 25°C (mM).

Table S4 TF quantum yields ($\Phi \pm 0.02$) of hGKRP-WT and hGKRP-P446L under different medium conditions at 20°C

The buffer contained 20 mM phosphate, 50 mM KCl, 1 mM EDTA and 1 mM DTT (pH 7.2).

Medium	hGKRP-WT Φ Fl. λ_{max}(nm)	hGKRP-P446L Φ Fl. λ_{max}(nm)	Trp-NATA Φ Fl. λ_{max}(nm)
Buffer	0.22 (332)	0.24 (332)	0.14 (350)
Urea (8 M)	0.14 (340)*	0.15 (340)*	–

*n^2 (urea)/n^2 (H_2O) = 1.103.

Table S5 $\Delta G(H_2O)$ value from urea denaturation curves for GK-WT and hGKRP-WT and their mutants in buffer at 20°C

The buffer contained 20 mM phosphate, 50 mM KCl, 1 mM EDTA and 1 mM DTT (pH 7.2).

Protein	$\Delta G(H_2O)$ ($\pm$0.05 kcal/mol)
GK-WT	1.63
GK-S263P	1.33
GK-M298K	1.38
GKRP-WT	1.56
hGKRP-P446L	1.55

© 2014 The Author(s)
This is an Open Access article distributed under the terms of the Creative Commons Attribution Licence (CC-BY) (http://creativecommons.org/licenses/by/3.0/) which permits unrestricted use, distribution and reproduction in any medium, provided the original work is properly cited.

Table S6 Curve fitting analysis of hGKRP-WT/hGKRP-P446L and GK-W99R/W167F/W257F interactions in the absence and presence of F6P at 20 °C measured by the TF of hGKRP

ΔF is the relative fluorescence change at 340 nm.

Agents	ΔF	$S_{0.5}$ (μM)	H	χ^2
hGKRP-WT and GK-W99R/W167F/W257F	0.29	1.22	1.74	7.4×10^{-5}
	0.39	1.85	1	18×10^{-5}
hGKRP-WT, F6P and GK-W99R/W167F/W257F	0.31	1.20	1.71	1.7×10^{-5}
	0.43	1.84	1	15×10^{-5}
hGKRP-P446L and GK-W99R/W167F/W257F	0.44	2.05	1.86	4.2×10^{-5}
	0.82	5.23	1	28×10^{-5}
hGKRP-P446L , F6P and GK-W99R/W167F/W257F	0.44	1.66	1.31	29×10^{-5}
	0.55	2.45	1	29×10^{-5}

REFERENCES

1 Monod, J., Wyman, J. and Changeux, G. P. (1963) On the nature of allosteric transition: a possible model. J. Mol. Biol. **12**, 88–119

2 Koshland, D. E., Nemethy, G. and Filmer, D. (1966) Comparison of experimental binding data and theoretical models in proteins containing subunits. Biochemistry **5**, 365–385

3 Changeux, G. P. (2011) 50th anniversary of the word “allosteric”. Protein Sci. **20**, 1119–1124

4 Zelent, B., Odili, S., Buettger, C., Shiota, C., Grimsby, J., Taub, R., Magnuson, M. A., Vanderkooi, J. M. and Matschinsky, F. M. (2008) Sugar binding to recombinant wild-type and mutant glucokinase monitored by kinetic measurement and tryptophan fluorescence. Biochem. J. **413**, 269–280

5 Neet, K. E. and Ainslie, Jr, G. R. (1980) Hysteretic enzymes. Methods Enzymol. **64**, 192–226

Received 11 October 2013/3 February 2014; accepted 26 February 2014
Published as BJ Immediate Publication 26 February 2014, doi:10.1042/BJ20131363

6 Neet, K. E., Keenan, R. P. and Tippett, P. S. (1990) Observation of a kinetic slow transition in monomeric glucokinase. Biochemistry **29**, 770–777

7 Cornish-Bowden, A. (1995) Fundamental Enzyme Kinetics. Portland Press, London

8 Cornish-Bowden, A. and Cardenas, M. L. (2004) Glucokinase: a monomeric enzyme with postitive co-operativity, In Glucokinase and Glycaemic Diseases: From Basics to Novel Therapeutics (Matschinsky, F. and Magnuson, M. A., eds), pp. 125–134, Karger, Basel

9 Frieden, C. (1979) Slow transitions and hysteretic behavior in enzymes. Ann. Rev. Biochem. **48**, 471–489

10 Zelent, B., Odili, S., Buettger, C., Zelent, D. K., Chen, P., Fenner, D., Bass, J., Stanley, C., Laberge, M., Vanderkooi, J. M. et al. (2011) Mutational analysis of allosteric activation and inhibition of glucokinase. Biochem. J. **440**, 203–215

11 Matschinsky, F. M., Zelent, B., Doliba, N. M., Kaestner, K. H., Vanderkooi, J. M., Grimsby, J., Berthel, S. J. and Sarabu, R. (2011) Research and development of glucokinase activators for diabetes therapy: theoretical and practical aspects. Handb. Exp. Pharmacol. **203**, 357–401

12 Szlyk, B., Braun, C. R., Ljubicic, S., Patton, E., Bird, G. H., Osundiji, M. A., Matschinsky, F. M., Walensky, L. D. and Danial, N. N. (2013) A phosphor-BAD BH3 helix activates glucokinase by a mechanism distinct from that of allosteric activators. Nat. Struct. Mol. Biol. **21**, 36–42

13 Choi, J. M., Seo, M. H., Kyeong, H. H., Kim, E. and Kim, H. S. (2013) Molecular basis for the role of glucokinase regulatory protein as the allosteric switch for glucokinase. Proc. Natl. Acad. Sci. U.S.A. **110**, 10171–10176

14 Agius, L. (2008) Glucokinase and molecular aspects of liver glycogen metabolism. Biochem. J. **414**, 1–18

15 Lloyd, D. J., St Jean, Jr, D. J., Kurzeja, R. J. M., Wahl, R. C., Michelsen, K., Cupples, R., Chen, M., Wu, J., Sivits, G., Helmering, J. et al. (2013) Antidiabetic effects of glucokinase regulatory protein small-molecule disruptors. Nature **504**, 437–440

16 Hill, A. V. (1910) The possible effect of aggregation of the molecules of hemoglobin on its dissociation curves. J. Physiol. **40**, 389–403

17 Hill, A. V. (1969) Bayliss and Starling and the happy fellowship of physiologists. J. Physiol. **204**, 1–13

18 Matschinsky, F. M., Zelent, B., Doliba, N., Li, C., Vanderkooi, J. M., Naji, A., Sarabu, R. and Grimsby, J. (2011) Glucokinase activators for diabetes therapy: May 2010 status report. Diabet. Care **34**, S236–S243

© 2014 The Author(s)
This is an Open Access article distributed under the terms of the Creative Commons Attribution Licence (CC-BY) (http://creativecommons.org/licenses/by/3.0/) which permits unrestricted use, distribution and reproduction in any medium, provided the original work is properly cited.
